# Computational modeling of thermal combination therapies by magneto-ultrasonic heating to enhance drug delivery to solid tumors

**DOI:** 10.1038/s41598-021-98554-z

**Published:** 2021-10-01

**Authors:** Mohammad Souri, Madjid Soltani, Farshad Moradi Kashkooli

**Affiliations:** 1 Department of Mechanical Engineering, K. N. Toosi University of Technology, Tehran, Iran; 2 Department of Electrical and Computer Engineering, University of Waterloo, Waterloo, ON Canada; 3 Centre for Biotechnology and Bioengineering (CBB), University of Waterloo, Waterloo, ON Canada; 4 Advanced Bioengineering Initiative Center, Multidisciplinary International Complex, K. N. Toosi University of Technology, Tehran, Iran

**Keywords:** Cancer models, Computational biophysics, Computational models, Cancer microenvironment, Targeted therapies

## Abstract

For the first time, inspired by magnetic resonance imaging-guidance high intensity focused ultrasound (MR-HIFU) technology, *i.e.*, medication therapy and thermal ablation in one session, in a preclinical setting based on a developed mathematical model, the performance of doxorubicin (Dox) and its encapsulation have been investigated in this study. Five different treatment methods, that combine medication therapy with mild hyperthermia by MRI contrast ($\gamma -{Fe}_{2}{O}_{3}$) and thermal ablation via HIFU, are investigated in detail. A comparison between classical chemotherapy and thermochemistry shows that temperature can improve the therapeutic outcome by stimulating biological properties. On the other hand, the intravascular release of ThermoDox increases the concentration of free drug by 2.6 times compared to classical chemotherapy. The transport of drug in interstitium relies mainly on the diffusion mechanism to be able to penetrate deeper and reach the cancer cells in the inner regions of the tumor. Due to the low drug penetration into the tumor center, thermal ablation has been used for necrosis of the central areas before thermochemotherapy and ThermoDox therapy. Perfusion of the region around the necrotic zone is found to be damaged, while cells in the region are alive and not affected by medication therapy; so, there is a risk of tumor recurrence. Therefore, it is recommended that ablation be performed after the medication therapy. Our model describes a comprehensive assessment of MR-HIFU technology, taking into account many effective details, which can be a reliable guide towards the optimal use of drug delivery systems.

## Introduction

Classical chemotherapy for the treatment of solid tumors usually uses low molecular weight and small-sized (≤ 1 nm) cytotoxic drugs such as doxorubicin (Dox)^1^. Side effects of classical chemotherapy led to the development of a concept called nanomedicine to transport drugs to the target site through drug-loaded nanoparticles^2–6^. Meanwhile, liposomes with a large size (≥ 70 nm) have been more welcomed due to the high load capacity^7^. However, it is reported that only about 0.7% of nanoparticles are able to reach the target site based on the enhanced permeability and retention (EPR) effect, leading to their poor clinical translation^8^. On the other hand, the elevated interstitial fluid pressure (IFP), and the high density of the extracellular matrix (ECM) in the tumor microenvironment have also impaired the penetration depth of nanoparticles^9,10^. In order to bypass these impediments, the paradigm of intravascular triggered drug release has been suggested^11^. Lysolipid thermally-sensitive liposome (ThermoDox) is a promising nanocarrier for this operation^12^. ThermoDox can be very effective when it meets two basic needs: (1) long circulation with the minimal content release; (2) fast and efficient content release, ideally within seconds. A stimulus for rapid response is required for the drug release^13^. Temperature is known to be the most effective stimulus for the release of content, helping the burst release of the drug from the carrier by increasing the temperature beyond the melting point of the carrier in the tumor vascular network^14^.

Achieving the highest potential of a drug delivery scheme is having a real-time monitoring system to control drug release and observe drug concentrations at the target site. Magnetic resonance imaging (MRI) is a powerful imaging technique that has no harmful rays. Magnetic nanoparticles (MNPs), which are used as contrast agents in MRI, can accumulate in two types in tumor microenvironment; direct and intravenous injections^15^. MNPs can be injected directly into the tumor, which is an invasive procedure and carries a high risk of metastasis. This risk can be eliminated by intravenous injection, causing the uniform distribution of MNPs in tumor tissue. It has been reported that MNPs tend to accumulate and form clusters of MNPs after extravasation^16–21^. After giving enough time for the accumulation of MNPs, the tumor can be analyzed with clearer images. MNPs, on the other hand, can under an alternating magnetic field by relaxation mechanisms liberate heat and induce mild hyperthermia^22^. The generated heat can be used to release the drug from ThermoDox. Therefore, MNPs are an excellent candidate for both the diagnostic and therapeutic methods, *i*.*e*., theranostics (a new concept which involves the integration of therapeutics and diagnostics in a single platform). However, it is very difficult, if not impossible, to reach the tumor center due to poor vascular network function and poor perfusion. Therefore, an auxiliary concept is needed to damage the cells in central areas. High-intensity focused ultrasound (HIFU) is a non-invasive thermal procedure that can raise the temperature of the tumor center using focused ultrasound waves to such an extent that the central areas become irreversibly necrotic^23–25^. However, poor control of temperature, thermal dose, and time spans in this method have prevented clinical trials from reaching clinical endpoints. MRI-guidance HIFU (MR-HIFU) is a versatile operating system which can control thermal ablation using user-defined protocols. It may be used in a more personalized approach in cancer therapy^26^.

**Figure 1:**
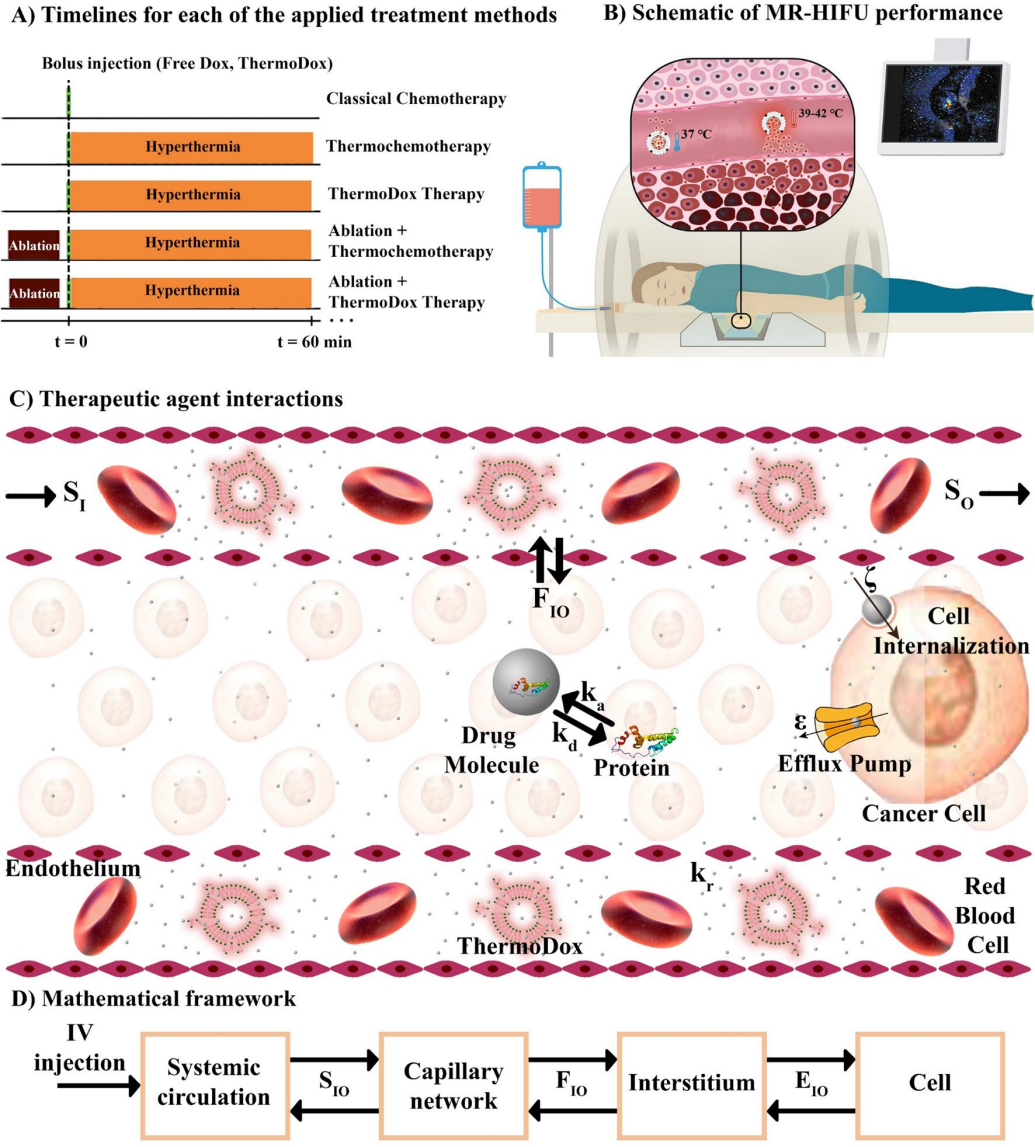
An overview of what is being done in this study; (**A**) Timelines of each all the applied treatment methods including classical chemotherapy, thermochemotherapy, ThermoDox therapy, ablation + thermochemotherapy, and ablation + ThermoDox therapy. In all methods, Dox is used as an anti-cancer therapeutic agent (adapted with permission from^29^). (**B**) Schematic of MR-HIFU which is used for hyperthermia and ablation. This is an advanced technology that allows the user to make the right decision by monitoring what is happening. (**C**) Schematic of interactions of therapeutic agents. Drug molecules can mainly bind to proteins in plasma which make them unable to reach their potential. It is assumed that only free drug enters the cell, on the other hand, the cell can expel them due to its multidrug resistant (MDR) properties. (**D**) Multi-compartment model of the present study. The current mathematical model is based on multi-compartment models that consider each biophysical environment as a compartment, and therapeutic agents are exchanged between these compartments. ${S}_{IO}$: Exchange of therapeutic agents between systemic circulation and capillary network, ${F}_{IO}$: Exchange of therapeutic agents between capillary network and interstitium,${E}_{IO}$: Exchange of therapeutic agents between interstitium and cell,${k}_{a}$: Association with protein, ${k}_{d}$: Dissociation with protein, $\varepsilon$: Cellular efflux functions, $\xi$: Cellular uptake functions.

Using in silico models, MR-HIFU thermal therapies with intravascular Dox delivery from ThermoDox have been investigated for the first time in a preclinical setting. We have developed a mathematical framework that describes the pharmacokinetics and pharmacodynamics of nano-drugs in great detail. In this work, the heat produced by the MRI contrast ($\gamma -{Fe}_{2}{O}_{3}$) is used for intravascular drug release from the carrier. The role of HIFU for tumor thermal ablation on the drug delivery process is also evaluated. This raises the main question in this study that is: Why, unlike other studies^26^ that have used MR-HIFU technology, HIFU has not been used for mild hyperthermia? Based on the literature^27^, the authors did not consider HIFU (single-element transducer) to be able to raise the tumor temperature evenly, so in most areas of the tumor, the drug is not released from the carrier. Therefore, in this study, hyperthermia caused by contrast MRI was taken, which is able to increase the tumor temperature relatively uniform. On the other hand, long-term use of ultrasound may cause the tumor motion^28^, which can be very challenging, so it has been used for ablation in a short time with high temperature. An overview of what is being done in this work is provided in Fig. 1.

## Results

The model consists of three separates physics; namely, therapeutic agent transport, heat transfer, and HIFU. Drug transport within interstitium is based on the convection–diffusion–reaction (CDR) equations. Heat transfer is based on the advanced energy equation in biological tissue (bio-heat model), which arises from the energy generated by the magnetic field and the propagation of sound. The magnetic field is also used for mild hyperthermia. Due to the fact that the temperature is considered to be less than 43 ℃ in mild hyperthermia^13^; the strength, frequency, and size of MNPs in alternating magnetic field (AMF) should be in accordance with the selection conditions (Supporting Information Table S1). To this aim, MNPs size of 18 nm under an AMF with a power of 13 $(kA\cdot {m}^{-1})$ and a frequency of 300 kHz has been used. The tissue is exposed to mild hyperthermia for 1-h (Supporting Information, Fig. S1). Additionally, sound propagation is simulated based on the Helmholtz model. A single-element transducer with a power of 30 W and a frequency of 1.44 MHz has been used to ablate the tumor under acoustic wave propagation for 60 s.

### Classical chemotherapy + Thermochemotherapy

**Figure 2:**
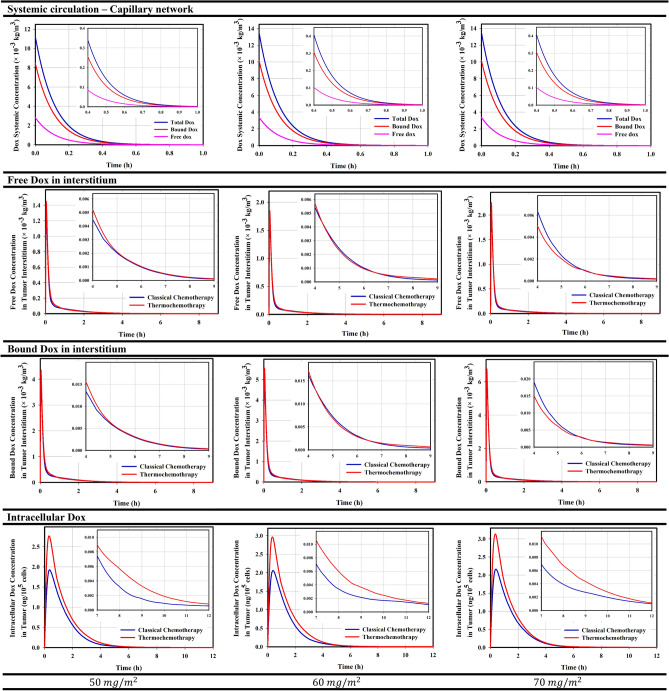
Temporal distribution of drug at different compartments during drug delivery; Increasing the injected dose enhances the concentrations of free and bound drugs at each compartment. Variation of the free and bound drugs in the microvessels depend on the changes in the injected drug. In the extracellular space, the variation of the bound drug is a function of the free drug. The peak intracellular concentration of the drug occurs about 20-min after the peak of free drug in extracellular space due to the time of crossing the transmembrane. The application of hyperthermia causes a change in the concentration of free and bound drug concentrations during drug delivery process due to change in permeability and transmembrane rate.

The low molecular weight combined with the small size of Dox make it easy to extravasate from microvessels and enter the interstitium to damage cell DNA. Mild hyperthermia has special benefits along with chemotherapy known as thermochemotherapy. Enhancing vascular permeability as well as increasing temperature improve the transvascular of therapeutic agents. At the cellular level, hyperthermia also alters biological pathways and increases cellular uptake of the drug. Figure 2 shows the Dox concentrations at different compartments for delivery of classical chemotherapy and thermochemotherapy in three permissible doses. It is clear that increasing the dose has led to a linear increase in Dox concentration in different compartments. According to reports, only 25% of the injected drug remains free in circulatory system and has a chance to enter the tissue interstitium (Supporting Information, Eqs. 7 and 8). After extravasation, the drug accumulates in the extracellular space, in which a part of the free drug also binds to the protein. High concentration in the tumor interstitum occurs due to the higher rate of transvascular exchange than the transmembrane transport. Comparison of free- and bound drug concentrations in classical chemotherapy and thermochemotherapy in ECM shows that the concentration level of free drug followed by the bound drug is higher in thermochemotherapy than those in classical chemotherapy. This is due to biological changes which happens in response to rising temperature. Increasing the temperature improves the vascular permeability (Supporting Information, Eq. 86), indicating an increase in the vessel-wall pore size. Although this issue improves drug accumulation, it also increases the chance of the drug leaving the tissue into the bloodstream (Supporting Information, Fig. S2). In fact, the drug transport from the tumor tissue into the microvessel happens due to the higher level of drug concentration within the tumor than the microvessel. Additionally, the increase in tumor interstitial concentration occurs due to the higher rate of transvascular exchange than the transmembrane transport. On the other hand, poor diffusion due to high IFP prevents deep penetration; so, it inevitably enters the surrounding healthy tissues or microvessels.

**Figure 3:**
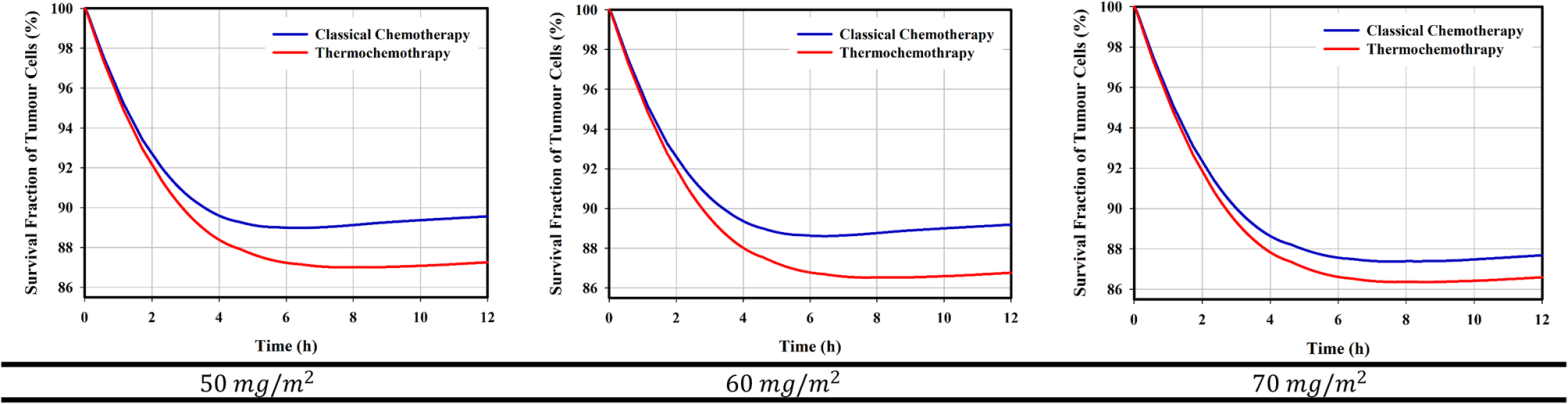
Cell mortality over time; In general, increasing the injected dose enhances the cell-death rate. Additionally, by applying mild hyperthermia due to the increase in transmembrane rate, the cell nucleus is exposed to much free drugs, so its chance of death enhances. Therefore, hyperthermia increases cell-death and delays the tumor growth onset.

Increasing the tissue temperature changes the membrane structure of the cell, improving the passive transport of the drug through the membrane by diffusion mechanism. The temperature of tissue reaches 42.6 °C in some areas, leading to a 2.4-fold increase in the exchange rate of transmembrane (Supporting Information, Eq. 87). Although an increase in temperature results in an increase in drug efflux from the cell, in the final case it also leads to an increase in drug accumulation within the cell. Temporal changes in intracellular drug concentrations are reported in Fig. 2, in which the intracellular concentration drops to zero after 5-h. This is happened due to the poor half-life of Dox in the circulatory system after intravenous Dox injection. Figure 2 also shows that the half-life of Dox, in this case, is lower than 30-min. This is a worrying problem for therapeutics response, the results of which are reported in Fig. 3. It is known that in the first 6-h post-injection, when the cell is exposed to an acceptable concentration of drug for toxicity, in all three injected doses, more than 11% of the tumor cells are destroyed; because in this period, the rate of cell mortality dominates cell proliferative. Tumor cells begin to proliferate at a rate of $3\times {10}^{-6} \left({s}^{-1}\right)$ after 6-h post-injection, when the concentration of intracellular drug does not overcome cell proliferation. This agrees with the in vivo results of Hijnen et al.^26^, in which they found that tumor growth began on the same day in the free drug injection group. Figure 3 also reports that increasing the dose and applying hyperthermia, in addition to enhancing cell death, can delay tumor growth onset.

The amount of drugs that are extravasated into the tissue through the microvessels depends on the characteristics of the tissue, including the density of the microvessels. Thus, in any region where the tissue density is higher, the drug accumulates more in that area. Specifically, drugs mainly accumulate near the microvessels in the proliferation zone. The dominant mechanism in the tumoricidal penetration depth of the drug is diffusion because the convection mechanism can be negligible due to the very small value of IFV in tumor interstitium. However, high IFP prevents deep penetration, causing the drug accumulation near the microvessels and at the proliferation zone (Supporting Information, Fig. S3).

### ThermoDox therapy

Problems such as side effects, low levels of drug concentrations within the tumor interstitium, and low rate of cell killing in classical chemotherapy have led to the use of ThermoDox. In response to heat, ThermoDox releases its cargo in the heated zone. Mild hyperthermia according to defined conditions has been used to heat the tumor. Here, 100 nm ThermoDox is used, which is able to release its payload within the tumor microvascular network at a temperature higher than the melting point of the lipid layer ($39-41^\circ{\rm C}$) in less than $60 s$.

**Figure 4:**
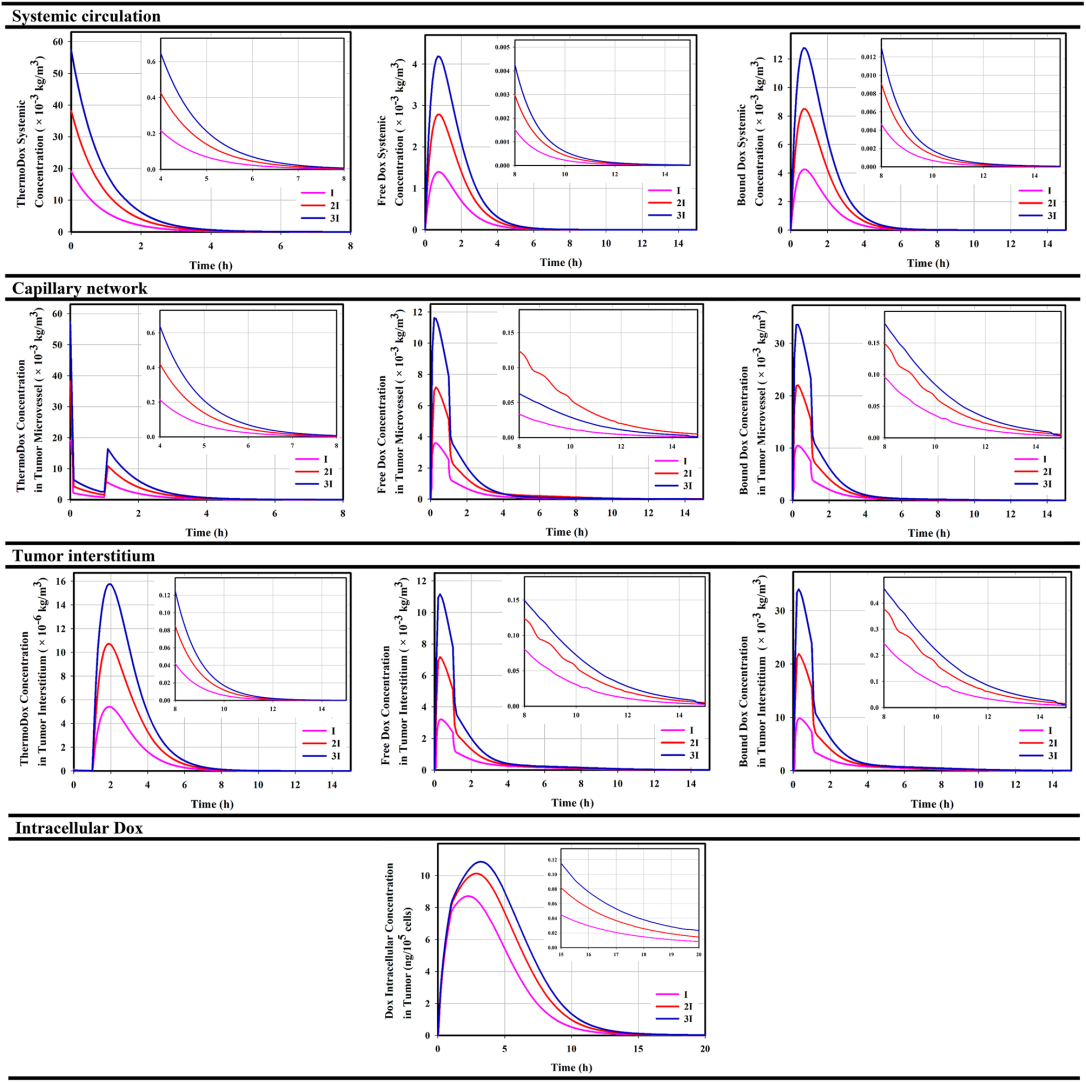
Temporal distribution of drug at different compartments during ThermoDox-mediated delivery; Increasing the injected dose of ThermoDox enhances the concentration of therapeutic agents at each stage $\left( {I = 0.0191\frac{kg}{{m^{3} }}, 2I = 0.0382 \frac{kg}{{m^{3} }}, 3I = 0.0573 \frac{kg}{{m^{3} }}} \right)$. The concentration of free and bound drugs in the capillary network is more than twice that of systemic plasma, which rapidly crosses the vessel wall and enters the tumor interstitium. The bound drug has similar variation with the free drug, but nearly three times greater. Concentration in intracellular compartment could have more drug due to the high extracellular concentration of drug.

Encapsulation of the drug reduces its nonuniform distribution, so higher doses of the drug can also be used for therapeutic purposes. To this aim, $$100 mg/{m}^{2}$$ and $$150 mg/{m}^{2}$$ Dox, which are loaded at $$0.0382 mg/{m}^{3}$$ and $$0.0573 mg/{m}^{3} $$ThermoDox, have been also used in addition to $$50 mg/{m}^{2}$$. ThermoDox is unstable at body temperature and releases some of the cargo rapidly in contact with the blood, which shortens its half-life, in addition to clearance. The drug released in the systemic plasma remains free or bound, which can be exchanged with various organs. Figure 4 shows the temporal variations in the levels of ThermoDox as well as free and bound drugs at different stages of ThermoDox-mediated drug delivery. Increasing the injected dose linearly affects the concentration of therapeutic agents. Thus, *3I* produces the highest concentration $$\left(I=0.0191\frac{kg}{{m}^{3}}\right)$$. ThermoDox in the capillary network of the tumor is exposed to a temperature higher than its melting temperature, which causes the burst release, resulting in a sharp decrease in the concentration of ThermoDox during hyperthermia. This leads to a high concentration of free drugs in the microvessels. Most of the released drug binds to the plasma protein, a few part is uptaken by endothelial cells, leading to vascular damage. Eventually, the remaining part enters the tumor. ThermoDox, free and bound drugs are exchanged with systemic plasma based on perfusion. However, due to the ratio of the volume of microvessel plasma of tumor to volume of systemic plasma ($${V}_{Tp}/{V}_{Sp}$$), variations in concentration of microvessel plasma of tumor do not have a significant effect on systemic plasma (Supporting Information, Fig. S4).

The large gap between the vascular endothelial cells of the tumor allows some ThermoDoxes to enter the tumor. However, in the first hour post-injection, due to the rapid release of the drug into the capillary network of the tumor, virtually no nanoparticles have a chance of entering the tumor except after hyperthermia. An important aspect of the intravascular release is the very high local concentration of the drug in a relatively small volume that is not uptaken by the cancer cells in a timely manner, causing some of them to return the tumor microvessels after heating (Supporting Information, Fig. S5). Intravascular drug release causes the concentration of free drug in the interstitium of the tumor increases more than 2.6 times compared to classical chemotherapy. However, Hijnen et al.^26^ reported 2.9 times for this value in their in vivo study. The difference is due to the fact that the rate of protein binding in the tumor microvessels and systemic circulation is considered equal in our study, whearas because of the rapid release of the drug, this rate must be lower.

**Figure 5:**
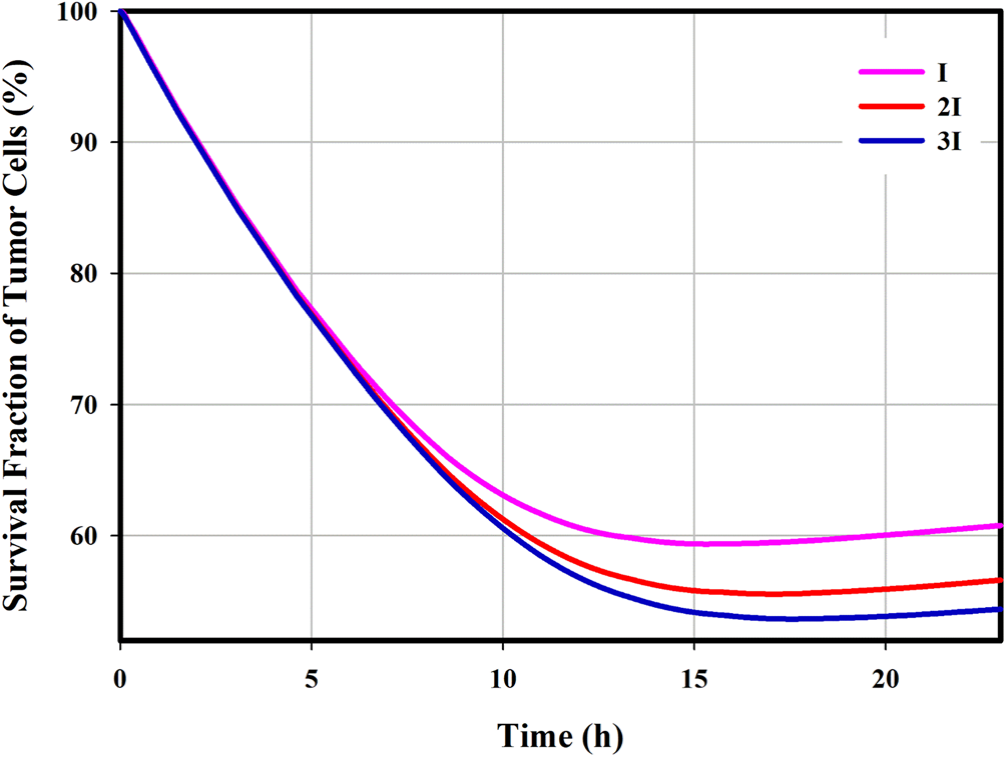
Cell mortality over time; Increasing the concentration enhances cell-death in a certain volume of tumor $\left( {I = 0.0191\frac{kg}{{m^{3} }},2I = 0.0382 \frac{kg}{{m^{3} }}, 3I = 0.0573 \frac{kg}{{m^{3} }}} \right)$.

Encapsulation of the drug increases its bioavailability for a long time. Therefore, cancer cells are exposed to the drug for a longer period of time. On the other hand, the high concentration of the drug in the tumor interstitium causes the internalized drug have a higher peak than classical chemotherapy. According to the temporal distribution of intracellular concentration in Fig. 4, it is clear that the internalized drug is at a relatively high level for about 15-h. As shown in Fig. 5, this causes mortality due to toxicity to overwhelmed cell-proliferation over a longer period of time, resulting in the death of more than 40% of cells. Although increasing the dose increases cell-death, eventually after 15-h post-injection, cell-proliferation overcomes cell-death and the tumor begins to grow. The contribution of anti-cancer drug toxicity to cell-degradation is negligible. By examining the rate of physiological degradation and cell-proliferation (Supporting Information, Eq. 57), it can be estimated that after 80-h, the cell-density increases to more than 10%, resulting in a significant increase in tumor volume. This result is also consistent with the in vivo study of Hijnen et al.^26^. They found that in the combination of liposomes and mild hyperthermia for intravascular release, it takes less than four days for the tumor volume to increase significantly in size after treatment.

High concentrations of the drug, relying on the diffusion mechanism, can led to deeper tumor penetration as well as cell-damage in that area. Howerver, thermoDox accumulates at the tumor periphery due to its poor diffusion coefficient (Supporting Information, Fig. S6). Drug release within the normal microvessel, is not significant. In fact, therapeutic agentswithin the normal microvessel are consistent with systemic circulation. ThermoDox is not able to penetrate the normal tissue because of its high ratio of ThermoDox size to the vessel-wall pore size of normal tissue (5–6 nm). However, due to the exchange of normal tissue with tumor tissue, tumor-driven ThermoDoxes can enter normal tissue. The trend of ThermoDox variations in normal tissue is consistent with tumor tissue but with lower orders, because the level of ThermoDoxes decreases in the tumor interstitium due to the drug release under hyperthermia (Supporting Information Fig. S7).

### Ablation therapy + Medication therapy

**Figure 6:**
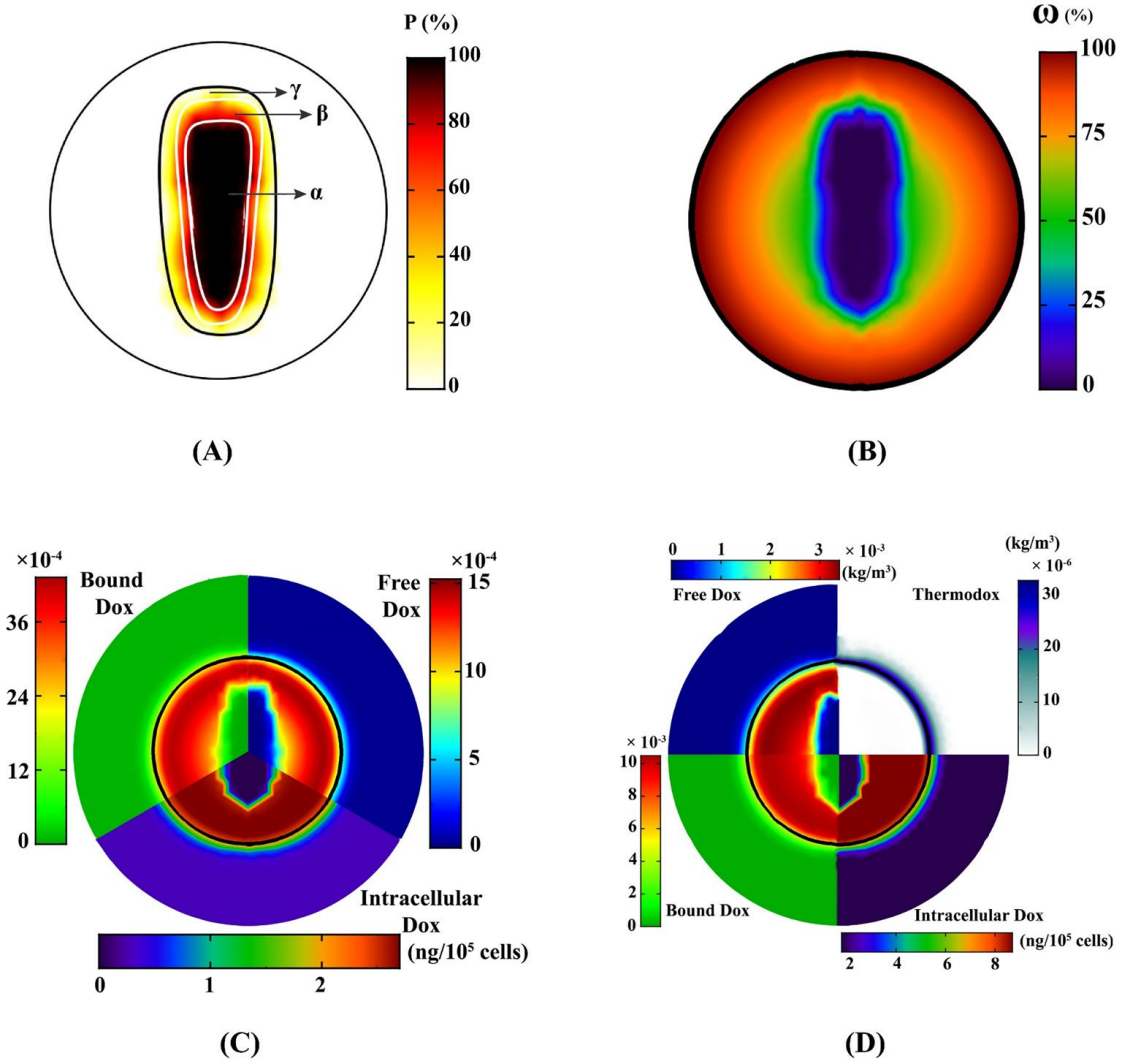
Ablation and medication therapy; (**A**) Thermal ablation forms three regions: coagulative necrosis (α), sublethal necrosis (β), and the region in which cells are not affected (γ). (**B**) Perfusion damage due to thermal ablation; the perfusion is zero in the α region, the perfusion yield is less than 25% in the β region, and the perfusion yield is half in the γ region. This contour indicates perfusion and vascular density. (**C**) Spatial distribution of the drug in the combination of thermochemotherapy and thermal ablation. (**D**) Spatial distribution of the therapeutic agents in the combination of ThermoDox therapy and thermal ablation.

Although ThermoDox can affect large volumes of tumors by releasing large concentrations of the drug, accessing to the tumor center is still a challenge, especially for the most-commonly used low doses. In fact, poor vascular density in hypoxic regions in the tumor center, poor diffusion coefficient due to high IFP, and infiltration resistance due to convection mechanism (with less impact) are the most important reasons of poor accumulation of therapeutic agents in the tumor center. After the medication therapy period, the central cells of tumor can proliferate and the tumor will recur after a while; therefore, a solution must be found for this problem. Thermal ablation is the most common method that can kill center cells of tumor by increasing the temperature. In fact, thermal ablation is used for an area of the tumor that is deprived of sufficient drug concentration. HIFU is a new technology that is conceptually based on high-temperature-based ablation. To increase the temperature, it uses ultrasound beams which focus them at a point that is the tumor center in this study (Supporting Information Fig. S8). For a short time ($$60 s$$), it raises the temperature by more than $$65^\circ{\rm C} $$ (Supporting Information Fig. S9), resulting in coagulative necrosis. Heat-ablated lesions form three regions: (1) the central region, which is immediately beyond the application tip and undergoes coagulative necrosis due to thermal ablation ($$\alpha $$); (2) the transitional region of sublethal necrosis occurs through the thermal conduction of the central region, which is apoptotic or recovering from reversible injury ($$\beta $$); and (3$$)$$ the third region, which has a temperature of less than $$45^\circ{\rm C} $$, although the microvessels are vulnerable in this region, but the cells are not damaged for 60 s, *i.e*., cells are not affected by ablation ($$\gamma $$) (Fig. 6A).

An increase in temperature of more than $43^\circ{\rm C}$ causes damage to the tumor microvessels and affects perfusion (Fig. 6B). Therefore, the delivery of therapeutic agents to the target site is difficult. Thermal ablation of the tumor can be done before or after medication therapy. If the ablation is applied post-medication; *i.e.*, after the end of the thermochemotherapy and ThermoDox therapy, the tumor center that has not been exposed to the drug can be targeted. Indeed, thermal ablation occurs first and medication therapy begins immediately. Here, $50 mg/{m}^{2}$ of Dox is used in both methods. Figure 6C shows the spatial distribution of the drug in the combination of thermal ablation and thermochemotherapy. In thermochemotherapy, the level of free and bound drugs is still high in the proliferative region. However, drug molecules, relying on the diffusion mechanism, are able to enter the ablated zone at lower orders. Practiccally, this area acts as a reservoir for drugs that have been deprived of therapeutic function. However, due to the pressure gradient, they can eventually leave this region. The concentration of the internalized drug in the proliferative region is high, but in the $\beta$ region, where the cells are regenerating, the concentration of free and internalized drugs is very low and will not be able to prevent cell-proliferation significantly. Additionally, there is virtually no cell for internalization in the $\alpha$ region. Events in ThermoDox therapy and tumor ablation are also prevalent. Figure 6D shows that ThermoDoxes are still accumulated at the tumor boundary, while free and bound drugs have much higher concentration levels in the proliferative region than other regions, leading to high intracellular concentration. There is still the challenge of cell-proliferation in the $\beta$ region, although there is a higher drug concentration than in the thermochemotherapy in this region.

## Discussion

This study is inspired by the performance of MR-HIFU technology, which is a powerful tool for cell-death by thermal ablation. Despite the normal tissues surrounded the tumor tissue, complete eradication of the tumor volume is very difficult, because it may also affect normal tissue. Therefore, a combination of mild hyperthermia and ablation has been suggested. In this study, in a preclinical mathematical framework, local drug delivery either in its free or in encapsulated forms in combination with mild hyperthermia was used as a non-invasive method against tumor. Therapeutically, the comparison between thermochemotherapy and classical chemotherapy (Fig. 3) shows interesting results. The local increase in temperature can improve the accumulation of drugs in the tumor interstitium by enhancing the transvascular exchange rate. It also stimulates cell-membranes to increase transmembrane rates, which improves the therapeutic response. Another important point shown in this study is the increase in penetration depth due to the high concentration gradient. This issue have also shown in previousely-published in vivo studies^11,30^ by distancing the drug from the microvessels and reaching farther cells. However, delivery of therapeutic agents to the tumor center is very difficult. Ablation strategy to necrose an area that is not exposed to the drug can also be an interesting idea. Results showed that if ablation occurs first, the region around the necrotic cores is associated with vascular shutdown, which prevents accumulation of sufficient drug concentrations in this area to target cell-proliferation. On the other hand, part of the cancer cells may migrate to the necrotic region^31^; so, it is understandable that these areas can be a source for tumor regrowth. Therefore, it is recommended that ablation be performed after medication therapy. Despite the delay in tumor growth in different treatment methods, complete eradication was not achieved for each treatment scheme. To further improve the therapeutic efficacy, depending on the exact clinical application as well as the specific condition of each patient, preparing a suitable treatment schedule including the therapeutic dose, drug combinations, and the timing of successive injections—can be considered. Although the results of Figs. 3 and 5 demonstrate that to achieve a better therapeutic outcome, the bioavailability time is more important than the peak of drug concentration. Consecutive treatments of this scheme should include multiple hyperthermia-mediated drug delivery sessions, and finally thermal ablation in the last cycle of medication therapy.

Despite all issues with improving the drug bioavailability in the tumor interstitium, another challenging issue is Dox binding to the protein, an issue that has not been carefully investigated or even addressed. Despite all the interesting ideas such as the use of ThermoDox, more than 70% of the drug is still bound to the protein. However, binding is known as a drug reservoir, because the drugs can be unbinding from the protein over time and remain free to reach the target. To increase bioavailability, it is better to reduce the binding rate of drug, but not in a way that create side effects. What can be concerning is the high concentration of the drug being washed out the tumor border to the surrounding normal tissue, which weakens the therapeutic potential of intravascular release. Therefore, it is suggested that release into the tissue (extravascular-triggered release) be done with greater penetration depth.

The effects of conventional chemotherapy and ThermoDox have been well-studied in the literature. However, how drugs are distributed and identified, as well as their impact on cell-death is not intuitive, and it is very difficult to elucidate experimentally. The present study is the development of a mathematical model that predicts the success rate of reaching the therapeutic drug to target site by considering the major factors influencing the transport of therapeutic agents. Based on this model, the role of hyperthermia with classical chemotherapy and ablation with a hybrid medication therapy methods became clear as new ideas for cancer treatment. Comparison of the results with the in vivo study of Hijnen et al.^26^ shows that this model is very precise and logical. Due to the complexity and comprehensiveness, our model includes a large number of equations and parameters of tumor properties, treatment conditions, temperature, and other functions. The values of the parameters and formulations are extracted from the experimental data; so, they are qualitatively and quantitatively consistent with the experimental results. Therefore, the presented model is a step forward in achieving the goal of targeted oncology and can be used for further insight into drug delivery processes.

## Methods

**Figure 7:**
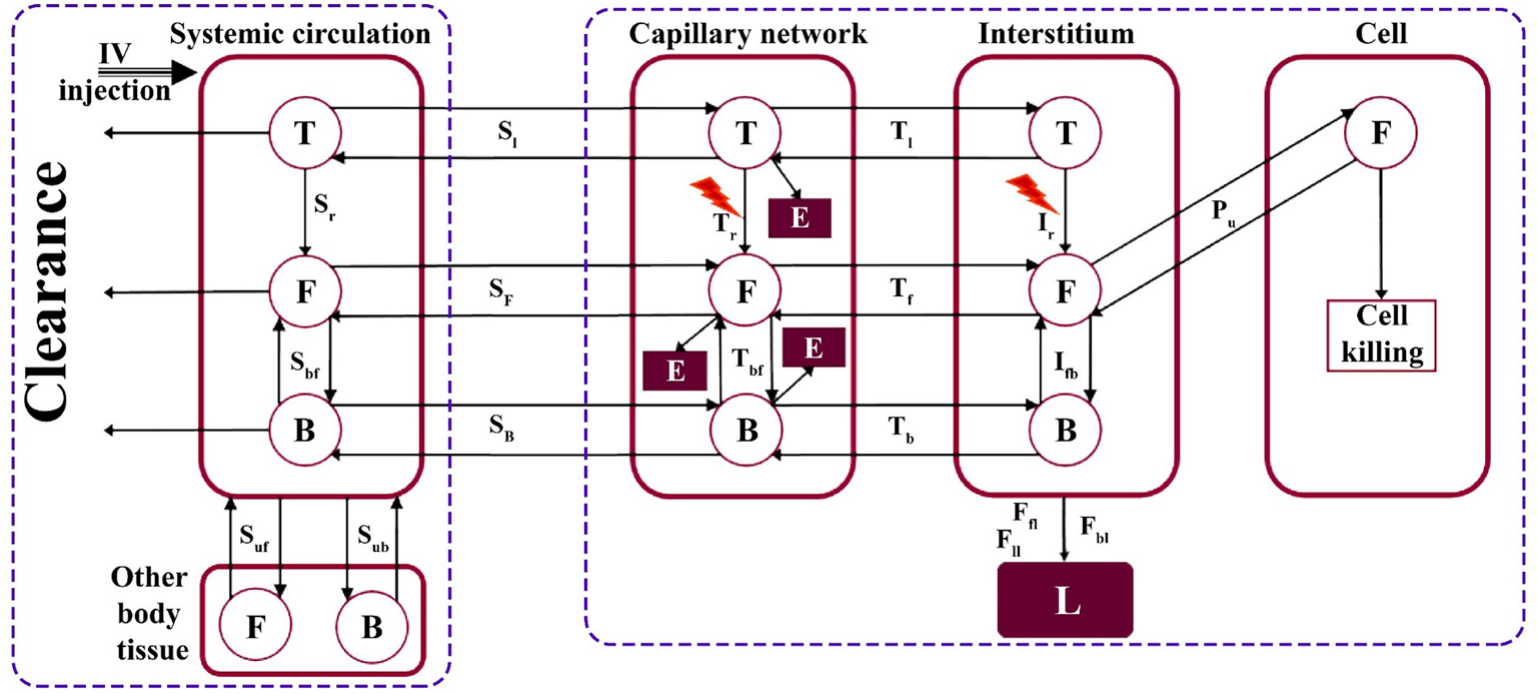
According to the drug transport processes, a multi-compartment model is presented, demonstrating the exchanges of therapeutic agents with tissues in the systemic circulation compartment, the elimination of therapeutic agents in the microvascular network due to immune systems and endothelial cell, excretion by the lymphatic system in the interstitium, and cellular uptake. T; ThermoDox, F; Free drug, B; Bound drug, L; Lymphatic system, E; Elimination.

A detailed description of the mathematical model and problem can be found in the Supporting Information file. This model is developed based on the previous models^32–35^ that bring the results closer to reality by considering many details. This is a multi-compartment model that calculates the concentration of the therapeutic agent in each compartment and its exchange rates with the adjacent compartments (Fig. 7). In classical chemotherapy, the systemic plasma and tissue plasma compartments are considered similar (Supporting Information Fig. S10).

The key assumptions considered in this study are as following:

- All cells are stationary, identical, and alive initially.
- Simulation duration is significantly shorter than the time-scale for tumor growth, and thereby the physiological parameters and geometry are independent of time.
- Based on density of microvessels, the tumor is divided into three areas: hypoxic, quiescent, and proliferative zones.
- In bolus injections, it takes less than a minute for the whole body to be exposed to the therapeutic agent^36^; so, it is assumed that the whole body is exposed to the injected concentration at zero time.
- ThermoDox is assumed to mix perfectly within plasma during injection.
- Only free Dox can enter the cell^37,38^, and when it enters the cell, it can damage DNA and kill the cell if it is not excreted by efflux pumps.
- It is assumed that MNPs (MRI contrasts) have already accumulated in the tumor. Accumulation of MNPs is considered as clusters for heat source under AMF in tumor tissue.
- The interactions of acoustic waves on MNPs have been ignored.

### Validation of model predictions

**Figure 8:**
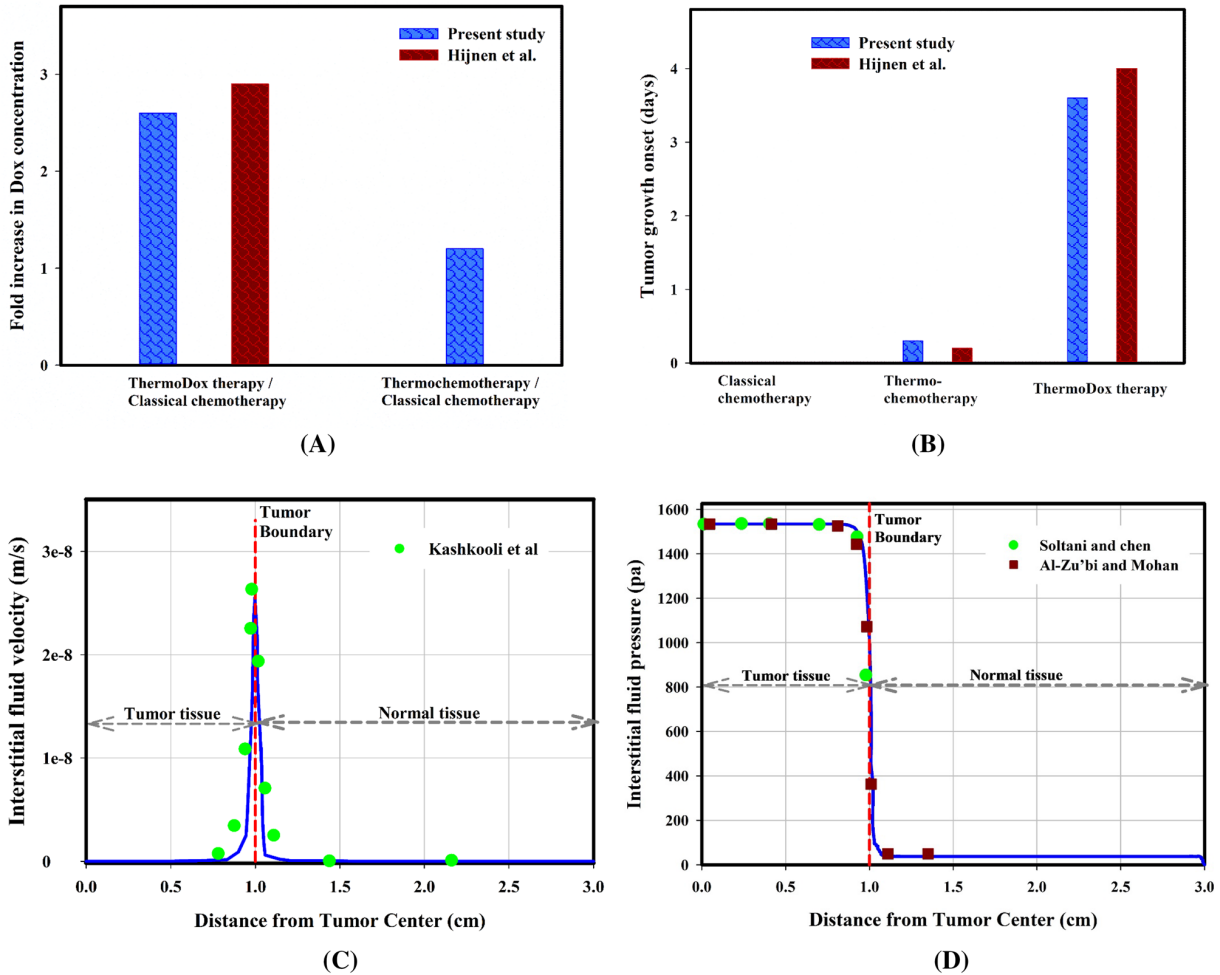
Validation cases; (**A**) Comparison of the fold increase in Dox accumulation in tumor tissue between the present mathematical model and the experimental data^26^, **(B**) Estimation of tumor growth onset in the day post-injection in mathematical and experimental models^26^ (in the mathematical model, tumor growth is based on about 10% increase in cell density), (**C**) IFV prediction in the present model has been compared with those of Kashkooli et al.^33^, and (**D**) IFP prediction in the present model has been compared with those of Soltani and Chen^43^, as well as Al-Zu’bi and Mohan^44^ models, which show high compatibility.

Measuring concentrations in preclinical in vivo models is very difficult, if not impossible. Therefore, sufficient experimental results are not available to validate the computational models and they are mostly verified qualitatively. Hence, the results of the present study are mainly validated by in vivo study of Hijnen et al.^26^ (Fig. 8A and B). Additionally, the comparison of the fluid flow in the tissue with previous numerical studies indicates the validity of the current model (Fig. 8C and D). Previously-published studies^32,34,39–42^ have also confirmed the equations, parameters, and assumptions considered in this study. As a consequence, the results of the current study are sufficiently reliable for further investigations.

## Supplementary Information

Supplementary Information.

## Data Availability

All data used for this study are available from the author upon request.
