# Supplementary material for: Computational modeling of thermal combination therapies by magneto-ultrasonic heating to enhance drug delivery to solid tumors
Source: Sci Rep. 2021 Oct 1;11:19539. doi: 10.1038/s41598-021-98554-z (PMC8486865; doi:10.1038/s41598-021-98554-z)
Supplement: Supplementary file 1 — Supplementary Information. [file 41598_2021_98554_MOESM1_ESM.docx]

**Supplementary Information**

**Computational modeling of thermal combination therapy by magneto-ultrasonic heating to enhance drug delivery to solid tumors**

Mohammad Souri^1^, Madjid Soltani^1,2,3,4,*^, Farshad Moradi Kashkooli^1^

^1^ Department of Mechanical Engineering, K. N. Toosi University of Technology, Tehran, Iran

^2^ Department of Electrical and Computer Engineering, University of Waterloo, Waterloo, ON, Canada

^3^ Centre for Biotechnology and Bioengineering (CBB), University of Waterloo, Waterloo, ON, Canada

^4^ Advanced Bioengineering Initiative Center, Multidisciplinary International Complex, K. N. Toosi University of Technology, Tehran, Iran

*Corresponding Author: Madjid Soltani

**Email:**  [msoltani@uwaterloo.ca](mailto:msoltani@uwaterloo.ca)

1. **Description of the mathematical model**

This document provides a detailed description of mathematical models. First, the mathematical equations related to the concentration distribution of doxorubicin (Dox) and lysolipid thermally sensitive liposome (ThermoDox) are described, which include the equations governing interstitial fluid flow, mass transfer in systematic plasma, and tissue plasma. Then, the heat transfer in the tissue and its microvessels in response to the magnetic and acoustic fields is investigated. The magnetic field is used for mild hyperthermia due to poor accumulation of magnetic nanoparticles (MNPs) in the tumor tissue, while High-intensity focused ultrasound (HIFU) is used to create irreversible necrotic tissue. Finally, the parameters related to drug transportthat are affected by various factors such as temperature are investigated.

- 1. **Interstitial fluid flow**

The interstitial fluid flow equations are solved to provide the basic biomechanical environment for drug transport. One of the first formulations to describe fluid flow in a porous medium is Darcy’s equation, which is totally appropriate for the fluid flow in tissues. This equation can be used for the relationship between interstitial fluid pressure (IFP), the interstitial fluid velocity (IFV), and is applicable to different biological tissues. Therefore, the fluid equation in a tissue can be defined as follows [1, 2]:

| $v=-\frac{\mu}{\kappa}\nabla p_{i}$ | (1) |
| --- | --- |

where $v$ and $p_{i}$ are the IFV and IFP, respectively. The mass conservation equation for interstitial fluid is given by [1]:

| $\nabla\cdot v=F_{v}-F_{ly}$ | (2) |
| --- | --- |

In Eq. 2, $F_{v}$ is the source terms that accounting for the gain of interstitial fluid from blood vessels and $F_{ly}$ is the sink terms that accounting for fluid absorption rate by the lymphatics. $F_{v}$ and $F_{ly}$ can be determined by Starling’s law;

| $F_{v}=K_{v}\frac{S}{V}\left[ p_{v}-p_{i}-\sigma_{T}(\pi_{v}-\pi_{i}) \right]$ | (3) |
| --- | --- |
| $F_{ly}=K_{ly}\frac{S_{ly}}{V}\left[ p_{i}-p_{ly} \right]$ | (4) |

where $K_{v}$ is the hydraulic conductivity of the microvessel wall, $\frac{S}{V}$ is the surface area of blood vessels per unit volume of tissue, $p_{v}$ is the vascular pressures. $\sigma_{T}$ represents the average osmotic reflection coefficient, $\pi_{v}$ is the osmotic pressure of the plasma, and $\pi_{i}$ is that of interstitial fluid.

The lymphatic drainage, $F_{ly}$, is related to the pressure difference between the interstitial fluid and
lymphatics. Lymphatic system is not considered for tumor tissue [3]. In Eq. 4, $K_{ly}$, $\frac{S_{ly}}{V}$ and $p_{ly}$are the hydraulic conductivity of the lymphatic wall, the surface area of lymphatic vessels per unit volume of tissue, and the intra-lymphatic pressure, respectively.

- 1. **Drug transport**

Drug administration routes and methods influence the kinetics of bio-distribution and elimination and hence determine the success of chemotherapy [4]. Modern pharmaceutical science offers numerous drug administration options with the aim of meeting particular needs in clinical cancer treatment.

- - 1. **Classical chemotherapy**

In classical chemotherapy, free Dox is injected. In this method, the free Dox circulates throughout the system. In addition to treatment of / reaching out the target site is able to access all organs.

- **Plasma pharmacokinetics**

The form of equations depends on the infusion mode. For bolus injection, the drug concentration is assumed to follow an exponential decay based on the plasma pharmacokinetics of Dox [5, 6].

| $C_{v}=DAe^{-\alpha t}$ | (5) |
| --- | --- |

Where $D$ is the dose of Dox, $A$ is the compartmental parameter, and $\alpha$ is the compartmental clearance rate, which is defined as:

| $\alpha=\frac{0.693}{t_{\frac{1}{2}}^{\alpha}}$ | (6) |
| --- | --- |

Where $t_{\frac{1}{2}}^{\alpha}$ is the (initial) half-life of Dox in plasma measured by Robert et al. [7] for a population of patients with breast cancer.

Free Dox in plasma can easily bind with proteins, such as albumin. Greene et al. [8] found that approximately 75±2.7% Dox is present in the bound form, and the percentage binding is independent of Dox and albumin concentrations. Hence for direct infusion, the free Dox ($C_{fp}$) and Dox-protein ($C_{bp}$) concentrations in plasma are given by

| $C_{fp}=0.25\times C_{v}$ | (7) |
| --- | --- |
| $C_{bp}=(1-0.25)\times C_{v}$ | (8) |

- **Free Drug transport in interstitium**

Drug transport is governed by the convection-diffusion-reaction (CDR) equations for the free drug and bound drug in the interstitial fluid [9]. Free Drug concentration in the interstitial fluid ($C_{fe}$) is described by

| $\frac{\partial C_{fe}}{\partial t}+\underset{\mathrm{Convection}}{\underbrace{\nabla\cdot\left( C_{fe}v_{i} \right)}}=\underset{\mathrm{Diffusion}}{\underbrace{D_{fe}\nabla^{2}C_{fe}}}+P_{f}$ | (9) |
| --- | --- |

Where $D_{fe}$ is the diffusion coefficient of free drug. The source term, $P_{f}$, is the net rate of
drug gained from the surrounding environment [9], given by

| $P_{f}=P_{fv}+P_{fb}+P_{u}$ | (10) |
| --- | --- |

Here $P_{fv}$, $P_{fb}$, and $P_{u}$ represent the net rate of drug gained from the blood/lymphatic vessels, association/dissociation with protein, and influx/efflux from tumor cells, respectively.

| $P_{fv}=F_{fp}-F_{fl}$ | (11) |
| --- | --- |

where $F_{fp}$ is the free drug exchange between the microvessel and interstitium, and $F_{fl}$ is the loss of drug to the lymphatic vessels per unit volume of tissue. Using the pore model [1, 10, 11] for transvascular exchange, $F_{fp}$ and $F_{fl}$ can be expressed as:

| $F_{fp}=F_{v}\left( 1-\sigma_{d} \right)C_{fp}+P_{fe}\frac{S}{V}(C_{fp}-C_{fe})\frac{{Pe}_{f}}{e^{{Pe}_{f}}-1}$ | (12) |
| --- | --- |
| $F_{fl}=F_{ly}C_{fe}$ | (13) |

where $\sigma_{d}$ is the osmotic reflection coefficient for the drug molecules, and ${Pe}_{f}$ is the permeability of vasculature wall to free drug. ${Pe}_{f}$ is the trans-capillary Peclet number defined as:

| ${Pe}_{f}=\frac{F_{v}\left( 1-\sigma_{d} \right)}{P_{fe}\frac{S}{V}}$ | (14) |
| --- | --- |

The net drug gained due to protein binding is governed by:

| $P_{fb}=k_{d}C_{be}-k_{a}C_{fe}$ | (15) |
| --- | --- |

Here $k_{a}$ and $k_{d}$ are the protein binding and dissociation rates, respectively.

The net drug gained due to cellular influx/efflux is governed by:

| $P_{u}=D_{c}\varepsilon-D_{C}\zeta$ | (16) |
| --- | --- |

Where $D_{c}$ is the tumor cell density and ζ and ɛ are cellular uptake and efflux functions.

- **Bound Drug transport in interstitium**

The convection-diffusion equation for bound drug ($C_{be}$) is similar to Eq. 9 except for the source terms [9].

| $\frac{\partial C_{be}}{\partial t}+\nabla\cdot\left( C_{be}v_{i} \right)=D_{be}\nabla^{2}C_{be}+P_{b}$ | (17) |
| --- | --- |

where $D_{be}$ is the diffusion coefficient of bound drug. The source term, $P_{b}$, is the net rate of
Dox gained from the surrounding environment [9], given by

| $P_{b}=P_{bv}-P_{fb}$ | (18) |
| --- | --- |

Here $F_{bv}$ is bound Dox gained from the blood/lymphatic vessels that it's defined as:

| $P_{bv}=F_{bp}-F_{bl}$ | (19) |
| --- | --- |

where $F_{bp}$ is the bound drug exchange between the microvessel and interstitium, and $F_{bl}$ is the loss of bound drug to the lymphatic vessels per unit volume of tissue. As in equations 12 and 13 for transvascular exchange of bound drug, $F_{bp}$ and $F_{bl}$ expressed as [9]:

| $F_{bp}=F_{v}\left( 1-\sigma_{d} \right)C_{bp}+P_{be}\frac{S}{V}(C_{bp}-C_{be})\frac{{Pe}_{b}}{e^{{Pe}_{b}}-1}$ | (20) |
| --- | --- |
| $F_{bl}=F_{ly}C_{be}$ | (21) |

where $\sigma_{d}$ is the osmotic reflection coefficient for the drug molecules, and ${Pe}_{b}$ is the permeability of vasculature wall to bound drug. ${Pe}_{f}$ is the trans-capillary Peclet number defined as:

| ${Pe}_{b}=\frac{F_{v}\left( 1-\sigma_{d} \right)}{P_{be}\frac{S}{V}}$ | (22) |
| --- | --- |

- **Intracellular Dox concentration**

Since only unbound drug can pass through the cell membrane [5, 6], the rate of cellular uptake is a function of free drug concentration in the interstitial fluid [9].

| $\frac{\partial C_{i}}{\partial t}=\zeta-\varepsilon$ | (23) |
| --- | --- |
| $\zeta=V_{max}\frac{C_{fe}}{C_{fe}+k_{e}\varphi}$ | (24) |
| $\varepsilon=V_{max}\frac{C_{i}}{C_{i}+k_{i}}$ | (25) |

where $V_{max}$ is the rate of transmembrane transport, ζ and ɛ are cellular uptake and efflux functions due to multidrug resistance pumps, $k_{e}$ and $k_{i}$ are constants obtained from experimental data, and φ is the volume fraction of extracellular space.

- - 1. **ThermoDox-mediated drug transport**

Cytotoxic drugs are encapsulated in NPs to decrease the risk of effects created by high concentration of drugs in healthy tissues, then intravenously administrated into the bloodstream [12]. When systematically administered, NPs are delivered to tumor regions via the circulatory system. ThermoDox have been developed for the purpose of intravascular release, although they may also enter the tissue interstitium. The drug released into the tissue plasma enters the tissue interstitium, and because it is released in the vascular, part of it inevitably enters the systemic plasma. In addition to binding to the protein, the drug in systemic plasma is uptake by other tissue body such as the heart, liver, kidney, spleen and muscles.

- - - 1. **Systemic plasma concentrations**

ThermoDox and the drug released in the systemic plasma interact with other organs. Therefore, their levels decrease over time.

- **ThermoDox concentration**

Pharmacokinetics of ThermoDox is described by one compartment, i.e., systemic plasma. Following an IV injection, the concentration of ThermoDox in the systemic plasma compartment ($\partial C_{LSP}$) is determined by [13]:

| $V_{Sp}\frac{\partial C_{LSP}}{\partial t}=-{CL}_{Lips}C_{LSP}V_{Sp}-S_{r}-S_{ls}+S_{lt}$ | (26) |
| --- | --- |
| $S_{ls}=F_{PT}C_{LSP}V_{Tp}$ | (27) |
| $S_{lt}=F_{PT}C_{LTP}V_{Tp}$ | (28) |
| $S_{r}={kr}_{37}C_{LSP}V_{Sp}$ | (29) |

Where $S_{ls}$, $S_{lt}$, and $S_{r}$ are the concentration of ThermoDox that enters the microvessel of tissue from the systemic plasma, the concentration of ThermoDox that enters the systemic plasma from the microvessel of tissue, and the concentration of drug released from ThermoDox in systemic plasma, respectively.${CL}_{Lips}$, ${kr}_{37}$ are the clearance rate of ThermoDox and release rate (at 37$℃$) in the systemic plasma, respectively. $V_{Sp}$, $V_{Tp}$ and $F_{PT}$ are the volume of systemic plasma, volume of microvessel plasma and plasma flow per microvessel plasma volume, which are functions of hematocrit and are defined as the following Eqs.

| $V_{Sp}=V_{BB}(1-H_{ct})$ | (30) |
| --- | --- |
| $V_{Tp}= V_{T}\times v_{Tp}$ | (31) |
| $v_{Tp}=$($V_{TV}(1-H_{ctt})$) | (32) |
| $F_{PT}=\omega(1-H_{ctt})/v_{Tp}$ | (33) |

Here $V_{BB}$, $H_{ct}$, $H_{ctt}$, $V_{T}$, $V_{TV}$ and $\omega$ are total blood volume in body, Hematocrit, Hematocrit for tissue microvasculature, volume of tissue, volume fraction of tissue vascular space, and blood perfusion rate, respectively.$v_{Tp}$ is volume fraction of microvessel plasma space.

- **Free drug** **concentration**

Free drug is present in the systemic plasma due to the instability of ThermoDox at body temperature. The drug is also exchanged with the target tissue and other body tissue. The concentration of free Dox in the systemic plasma ($C_{FSP}$) is defined as follows [13]:

| $V_{Sp}\frac{\partial C_{FSP}}{\partial t}=S_{r}+S_{bf}+S_{uf}-{CL}_{FS}C_{FSP}V_{Sp}-S_{fs}+S_{ft}$ | (34) |
| --- | --- |
| $S_{bf}=({k_{d}C_{BSP}-k}_{a}C_{FSP})V_{Sp}$ | (35) |
| $S_{uf}= {(k_{ft}C_{FSP}V_{BT}-k}_{fP}C_{FSP}V_{Sp})$ | (36) |
| $S_{fs}=F_{PT}C_{FSP}V_{Tp}$ | (37) |
| $S_{ft}=F_{PT}C_{FTP}V_{Tp}$ | (38) |

Here $S_{b}$, $S_{uf}$, $S_{fs}$, $S_{ft}$ are association/dissociation with protein in systemic plasma, drug exchange between the systemic plasma and other body tissue, the concentration of drug that enters the microvessel from the systemic plasma, and the concentration of drug that enters the systemic plasma from the icrovascular, respectively. ${CL}_{FS}$ and $V_{BT}$ are the clearance rate of free drug in the systemic plasma and the volume of body tissue.$k_{a}$ and $k_{d}$are the binding and dissociation rates, respectively.$k_{fp}$ and $k_{ft}$ are transfer constant drug from systemic plasma to body tissue and transfer constant drug from body tissue to systemic plasma, respectively.

- **Bound drug concentration**

Bound drug, like free drug, has its same interactions. The concentration of a bound drug in systemic plasma ($C_{BSP}$) is defined by the following Equations:

| $V_{Sp}\frac{\partial C_{BSP}}{\partial t}=+S_{ub}-S_{bf}-{CL}_{BS}C_{BSP}V_{Sp}-S_{bs}+S_{bt}$ | (39) |
| --- | --- |
| $S_{ub}= {(k_{bt}C_{BSP}V_{BT}-k}_{bP}C_{BSP}V_{Sp})$ | (40) |
| $S_{bs}=F_{PT}C_{BSP}V_{Tp}$ | (41) |
| $S_{bt}=F_{PT}C_{BTP}V_{Tp}$ | (42) |

Where ${CL}_{BS}$ is the clearance rate of bound drug in the systemic plasma. $S_{bs}$ and $S_{bt}$ are the concentration of bound drug that enters the microvessel from the systemic plasma and the concentration of bound drug that enters the systemic plasma from the microvessel, respectively. In Eq. 40 $k_{bp}$ and $k_{bt}$ are transfer constant bound drug from systemic plasma to body tissue and transfer constant Dox-protein from body tissue to systemic plasma, respectively. The values of these two parameters have not been reported in a study, but the author has estimated them based on changes in other parameters.

- - - 1. **Microvessel concentrations**

The microvessel of target tissue is linked to the systemic plasma via blood perfusion, which accounts for convective transport of ThermoDox and free drug between the whole body and tumor. The release of anticancer drug from ThermoDox in tumor plasma depends on temperature, which can be described by the first-order kinetics with the release rate constants ${kr}_{37}$ and ${kr}_{P}$ at the body temperature and mild hyperthermia upon heating, respectively. In the following, the concentration of therapeutic agents in microvessel is investigated.

- **ThermoDox** **concentration**

The concentration of ThermoDox in microvessel is determined due to the eliminate by the macrophage system and endothelial cell, the exchange between systemic plasma and interstitium, as well as the drug release, which is defined by the following Equations:

| $V_{Tp}\frac{\partial C_{LTP}}{\partial t}=-{CL}_{Lipt}C_{LTP}V_{Tp}-T_{r}-T_{l}-S_{lt}+S_{ls}$ | (43) |
| --- | --- |
| $T_{r}={kr}_{P}C_{LTP}V_{Tp}$ | (44) |
| $T_{l}= F_{lp}V_{Tp}$ | (45) |

Where $T_{r}$ and $T_{l}$ are the drug released under mild hyperthermia in tissue plasma and the ThermoDox transvascular exchange, respectively. ${CL}_{Lipt}$ is the elimination rate of ThermoDox in the microvessel.

$F_{lp}$ is ThermoDox exchange between the microvessel and interstitium that it's defined as:

| $F_{lp}= F_{v}\left( 1-\sigma_{l} \right)C_{LTP}+P_{L}\frac{S}{V}(C_{LTP}-C_{l})\frac{{Pe}_{l}}{e^{{Pe}_{l}}-1}$ | (46) |
| --- | --- |

Here $\sigma_{l}$ and $P_{L}$ are the osmotic reflection coefficient for the ThermoDox, and $P_{L}$ is the permeability of vasculature wall to ThermoDox. ${Pe}_{l}$ is the trans-capillary Peclet number for ThermoDox that it’s defined as:

| ${Pe}_{l}=\frac{F_{v}\left( 1-\sigma_{l} \right)}{P_{L}\frac{S}{V}}$ | (47) |
| --- | --- |

- **Free drug concentration**

The concentration of drug in microvessel is determined due to the clearance/uptake by the endothelial cells, the exchange between systemic plasma and interstitium, as well as the drug release of drug and association/dissociation with protein, which is defined by the following Equations:

| $V_{Tp}\frac{\partial C_{FTP}}{\partial t}=T_{r}-T_{f}+T_{bf}-{CL}_{ftp}C_{FTP}V_{Tp}-S_{ft}+S_{fs}$ | (48) |
| --- | --- |
| $T_{bf}= (k_{d}C_{BTP}-k_{a}C_{FTP})V_{Tp}$ | (49) |
| $T_{f}=F_{fp}V_{Tp}$ | (50) |

Where $T_{bf}$ and $T_{f}$ are association/dissociation with protein in tissue plasma and the free Dox transvascular exchange, respectively. ${CL}_{ftp}$ is the elimination rate of free drug in microvscular. $F_{fp}$ is defined as equation 12.

- **Bound drug concentration**

Bound drug also has the same interactions as free drug, which is defined as follows:

| $V_{Tp}\frac{\partial C_{BTP}}{\partial t}=-T_{b}-{CL}_{btp}C_{BTP}V_{Tp}-T_{bf}-S_{bt}+S_{bs}$ | (51) |
| --- | --- |
| $T_{b}=F_{bp}V_{Tp}$ | (52) |

Where ${CL}_{btp}$ is the elimination rate of bound drug in microvessel and $T_{b}$ is the bound drug transvascular exchange. $F_{bp}$ is defined as equation 20.

- - - 1. **Interstitium concentrations**

Drug concentration in tissue interstitium and ThermoDox transport can be described by the CDR equation. Upon entering tissue interstitium, free drugs are uptake by cells. Intracellular drugs can be pumped out of cells, most likely by membrane transporter proteins (e.g., P-glycoprotein), which could be significant in resistant tumor cells associated with overexpression of transporter proteins. In addition, binding of free drugs to interstitial proteins (e.g., albumin) should be accounted for.

- **Liposome concentration**

In the microvessel network of the tumor tissue, the distance between the endothelial cells is 50 to 70 times that of normal tissue, which causes some of the ThermoDoxes to enter the tumor tissue and release their load into the tissue. The distribution of ThermoDox in tumor tissue is defined by the following equation:

| $\frac{\partial C_{l}}{\partial t}+\nabla\cdot\left( C_{l}v_{i} \right)=D_{l}\nabla^{2}C_{le}-I_{r}+I_{lp}$ | (53) |
| --- | --- |
| $I_{r}={kr}_{P}C_{l}$ | (54) |
| $I_{lp}=F_{lp}-F_{ll}$ | (55) |

Where $D_{l}$ is the diffusion coefficient of free drug. $I_{r}$ and $I_{lp}$ are Dox released from ThermoDox in interstitium under mild hyperthermia and the ThermoDox exchange by vessel/lymphatic, respectively. $F_{ll}$, which is related to the removal of ThermoDox in the tissue by the lymphatic system, is defined by the following Eq:

| $F_{ll}=F_{ly}C_{l}$ | (56) |
| --- | --- |

- **Free drug** **concentration**

In ThermoDox-mediated drug delivery, the concentration of free drug within the tissue is in accordance with equation 9, although the difference is that the drug release term ($I_{r}$, Eq. 53) is added to equation 11.

- **Bound drug concentration**

In ThermoDox-mediated drug delivery, the concentration of bound drug within the tissue is in accordance with equation 17.

- **Intracellular** **drug** **concentration**

In ThermoDox-mediated drug delivery, the concentration of Intracellular drug is in accordance with equation 23.

- 1. **Pharmacodynamics model**

The change of tumor cell density with time is described by a pharmacodynamics model as given below [14]. This model is defined based on intracellular concentration over time, ie, the model believes that the therapeutic response is maximal when the cells are exposed to the drug for a longer time.

| $\frac{dD_{c}}{dt}=-\frac{f_{max}C_{i}}{{EC}_{50}+C_{i}}D_{c}+k_{c}D_{c}-k_{g}D_{c}^{2}$ | (57) |
| --- | --- |

The first term on the right-hand side represents the anticancer effect, where $f_{max}$ is the cell-kill rate constant and ${EC}_{50}$ is the drug concentration producing 50% of fmax. $k_{c}$ and $k_{g}$ are cell proliferation rate constant and physiological degradation rate, respectively. In this study, cell proliferation and physiologic degradation are assumed to have reached equilibrium at the start of each treatment.

- 1. **Bioheat transfer**

In localized heating in tumor therapy, both blood vessels and tissue including the tumor and its holding tissue are heated. Energy balances for tissue and blood are illustrated in Fig. S11.

The temperature (T) of tissue and blood can be calculated by solving the following energy balance equations [9, 15, 16]:

| $\rho_{t}c_{t}\frac{\partial T_{t}}{\partial t}={k_{t}\nabla}^{2}T_{t}-\underset{The heat sink term due to blood perfusion}{\underbrace{\rho_{bl}c_{bl}w_{bl}\left( T_{t}-T_{b} \right)}}+\underset{The heat generated by metabolism}{\underbrace{Q_{m}}}+\underset{The external power deposition term}{\underbrace{Q_{x}}}$ | (58) |
| --- | --- |
| $\rho_{bl}c_{bl}\frac{\partial T_{b}}{\partial t}={k_{bl}\nabla}^{2}T_{bl}-\underset{The heat sink term due to blood perfusion}{\underbrace{\rho_{bl}c_{bl}w_{bl}\left( T_{b}-T_{n} \right)}}+\underset{The heat source term due to tissue}{\underbrace{\rho_{bl}c_{bl}w_{bl}\left( T_{t}-T_{b} \right)}}+\underset{The heat generated by metabolism}{\underbrace{Q_{m}}}+\underset{The external power deposition term}{\underbrace{Q_{x}}}$ | (59) |

Where $\rho_{t}$, $c_{t}$ and $k_{t}$ are the density, heat capacity and thermal conductivity of the tissue, respectively. $\rho_{bl}$, $c_{bl}$ and $k_{bl}$ are the density, heat capacity and thermal conductivity of blood, respectively. $w_{bl}$ is the perfusion rate of blood flow.$Q_{x}$ is the heat produced by an external stimulus. In this study, external stimuli are magnetic field and ultrasound. The heat generated by these two stimuli is effective in the energy equation. But the magnetic field does not affect the blood energy equation because magnet clusters are assumed in the tumor tissue.

- 1. **Magnetic field-mediated hyperthermia**

Magnetic iron oxide nanoparticles (IONPs) such as maghemite (γ-Fe2O3) or magnetite (Fe3O4) are widely used as contrast agents for magnetic resonance imaging due to their chemical stability, lack of toxicity, and biodegradability. Intravenous administration of MNPs has minimally invasive and can potentially deliver magnetic nanoparticles to poorly defined, nonlocalized cancer tumors of various shapes and sizes, including small metastatic tumor growths. In addition to the noninvasive diagnostic characteristics of MNPs, such particles can produce thermal energy under the influence of an external alternating current magnetic field [17].

For purposes of analysis, the particle size is assumed to be within the range that characterizes relaxation loss as the dominant heating mechanism in this paper, so it will be considered that this is the only heating mechanism of MNPs. The thermogenesis mechanism of MNPs can be described in detail using Rosensweig’s theory in terms of proper particle size, in which the heat energy absorbed by MNPs is related to the frequency and amplitude of the AMF, and the properties of the MNPs [18]. The amount of heat generated by MNPs in tumor tissue used in the energy equation is defined as follows:

| $Q_{x}=\gamma M$ | (60) |
| --- | --- |

here $\gamma$ is the correction coefficient for the power dissipation, which is considered 0.55 [19]. The power dissipation of the MNPs into heat as a result of their interaction with an AMF that can be represented as [20]:

| $M=\pi\mu_{0}\chi_{0}H_{0}^{2}f_{m}\frac{2\pi f_{m}\tau}{1+{(2\pi f\tau)}^{2}}$ | (61) |
| --- | --- |

here $\mu_{0}$, $H$, and $f_{m}$ are the permeability of free space, magnetic field strength, and magnetic field frequency, respectively. For a safe application of magnetic hyperthermia to patients, it was experimentally found that the product of the frequency and the magnetic field amplitude ($H\times f_{m}$) should be smaller than $5\times{10}^{9} (A\cdot m^{-1}\cdot s^{-1})$ [21]. $\chi_{0}$ is the static equilibrium susceptibility that it's can be given as:

| $\chi_{0}=\chi_{i}\frac{3}{\xi}\left( \frac{1}{tan\xi}-\frac{1}{\xi} \right)$ | (62) |
| --- | --- |

Where $\chi_{i}$ is initial susceptibility, which is given as:

| $\chi_{i}=\frac{\mu_{0}\emptyset M_{d}^{2}V_{m}}{3k_{B}T}$ | (63) |
| --- | --- |

Where $M_{d}$, $k_{B}$, $V_{m}$ and $T$ are the domain magnetization, the Boltzmann constant, the volume of magnetic particles (excluding the volume of their coating), and the absolute temperature of MNPs, respectively.

In Eq. 63, $\xi$ is a dimensionless parameter of the magnetic-thermal quantity which is defined as follows;

| $\xi=\frac{\mu_{0}{\emptyset M}_{d}H_{0}V_{M}}{k_{B}T}$ | (64) |
| --- | --- |

The effective relaxation time τ, referred to in Eq. 62, is defined as follows;

| $\frac{1}{\tau}=\frac{1}{\tau_{N}}+\frac{1}{\tau_{B}}==\gg\tau=\frac{\tau_{B}+\tau_{N}}{\tau_{B}{\cdot\tau}_{N}}$ | (65) |
| --- | --- |

where $\tau_{B}$ is the Brownian relaxation time and $\tau_{N}$ is Neel relaxation time, which defined by equations 66 and 67, respectively.

| $\tau_{B}=\frac{3\eta V_{H}}{k_{B}T}$ | (66) |
| --- | --- |
| $\tau_{N}=\frac{\sqrt{\pi}}{2}\tau_{0}\frac{exp\Gamma}{\Gamma^{1/2}}$ | (67) |
| $\Gamma=\frac{K_{a}V_{m}}{k_{B}T}$ | (68) |

Where $\eta$, $\tau_{0}$, and $K_{a}$ are the dynamic viscosity of the fluid, time constant, and the anisotropy constant of MNP, respectively.$V_{H}$ is the hydrodynamics volume of the MNP that it's defined by:

| $V_{H}=\left( 1-\delta/R \right)^{3}V_{m}$ | (69) |
| --- | --- |

Here $\delta$ and $R$ are the surfactant layer thickness and particle radius, respectively.

In equations 63 and 64, $\emptyset$ is MNP volume fraction which relates to the number of the MNPs per unit volume $n$, and the radius of the MNP $R$. The MNP concentration ($n$) is based on the study of Wang et al [22] is ${10}^{22}(m^{-3})$. As spherical MNPs are considered in this paper, the volume fraction of MNPs can be expressed as

| $\emptyset=n\cdot\left( \frac{4}{3}\pi R^{3} \right)$ | (70) |
| --- | --- |

- 1. **HIFU-mediated hyperthermia**

HIFU therapy is a noninvasive ablation method in which ultrasound energy from an extracorporeal source is focused within the body to locally ablate tissue at the focus without damaging surrounding tissues [23, 24]. HIFU is most widely used to thermally ablate a variety of both benign and malignant tumors including uterine fibroids, prostate cancer, breast cancer, liver tumors, and other solid tumors that are accessible to ultrasound energy [25-27]. The heating of biological tissue by HIFU also contains the effect of acoustic nonlinearity [28, 29]. However, studies show that the non-linear wave propagation can be ignored if a focal intensity is within the range of $100\sim1000 W/{cm}^{2}$ [9, 30, 31]. In present study, the propagation of ultrasound in a medium is investigated by solving linear propagation of the pressure wave is given by the Helmholtz equation [32]:

| $\frac{1}{\rho c_{0}^{2}}\frac{\partial^{2}P}{\partial t^{2}}+\nabla\cdot\left[ -\frac{1}{\rho}\left( \nabla P-q_{d} \right) \right]=Q_{p}$ | (71) |
| --- | --- |

Where $\rho$ and $c_{0}$ are the density and the speed of sound, respectively. $Q_{p}$ and $q_{d}$ are possible acoustic monopole and dipole source terms, respectively, which are considered zero. $Q_{p}$ can be used to represent a domain heat source causing pressure variations or a user defined nonlinear contribution to the equations in the time domain (Westervelt equation [16]). $q_{d}$ source represents a domain volumetric force.

The pressure, P, can be expanded into harmonic components using the Fourier series:

| $P=psin\left( \omega t \right)==\gg P=pe^{-i\omega t}$ | (72) |
| --- | --- |

Solving Eq. 72 with this complex variable yields the Helmholtz equation:

| $\frac{\kappa^{2}}{\rho}p+\nabla\cdot\left[ \frac{1}{\rho}\left( \nabla p \right) \right]=0$ | (73) |
| --- | --- |

where $\kappa$ is the wave number that it's defined by;

| $\kappa=\frac{\omega}{c}+i\alpha_{ABS}$ | (74) |
| --- | --- |

here $\alpha_{ABS}$ is the absorption coefficient. $\omega(=2\pi f)$ is the angular frequency and $f$ is the frequency.

The power ($P_{W}$) of the acoustic wave has a relation with the transducer's operating parameters through Eq. (76) [24]:

| $P_{W}=2S\pi^{2}\rho_{w}fc_{w}{\Delta x}_{max}^{2}$ | (75) |
| --- | --- |

where $S$ is the transducer area, $\rho_{w}$ and $c_{w}$ are the density and the speed of sound of the medium that is in contact with the transducer aperture, here is the water between the transducer and the tissue. $\Delta x$ is the normal displacement of the transducer aperture and the transducer contact area oscillates harmonically with this displacement value and transmits the desired sound pressure. Therefore, the transducer power could be regulated by changing the amount of normal displacement.

In order to couple the pressure field to the temperature field, we need to estimate the thermal energy deposition associated with the absorption of ultrasonic waves. The following equation [33] describes the ultrasonic power deposition per unit volume:

| $Q_{x}=2\alpha_{ABS}I=2\alpha(\frac{P^{2}}{2\rho})$ | (76) |
| --- | --- |

- 1. **Thermal damage**

Although it is generally accepted that tissue damage is an outcome of several complex mechanisms, Thermal damage in the tissue model was predicted using the Arrhenius law [34]. The Arrhenius model assumes that thermally induced cell injury is a first order irreversible kinetics process and the reluctant cell survival can be calculated by using the following equations.

| $\Omega\left( t \right)=ln\left( \frac{C(0)}{C(t)} \right)=\int_{0}^{t} Ae^{\frac{-\Delta E}{RT(t)}}dt$ | (77) |
| --- | --- |

where $\Omega\left( t \right)$, is the degree of biological tissue damage, $C(0)$ is the initial concentration of healthy biological cells, $C(t)$ is the concentration of healthy biological cells remaining after thermal stimulation, $R$ is the universal gas constant, $A$ is a frequency factor for the kinetic expression, and $\Delta E$ is the activation energy of the thermal damage process, and $T$ is the instantaneous absolute temperature of the cells during thermal stress, which is a function of time, $t$. The parameters $A$ and $\Delta E$ are dependent on the type of tissue and have been characterized for normal breast tissues by Henriques and Moritz [34] and breast tumor tissues by Bhowmik et al [35]. The probability of tissue cell death, $P_{n}\left( \% \right)$, is then expressed as [36]:

| $P_{n}\left( \% \right)=100\times\left( 1-e^{-\Omega\left( t \right)} \right)$ | (78) |
| --- | --- |

1. **Model parameters**

Since the growth of tumor and normal tissues is ignored, all the geometric and transport parameters used in this study are assumed to be independent of time (According to the study time, which is 24h). Values adopted for these are summarized in Tables S2, S3, S4, and S5 at the end of this chapter for parameters related to the physiological parameters, parameters for doxorubicin, parameters for ThermoDox, and parameters of the MNPs $(\gamma-{Fe}_{2}O_{3})$, respectively. Justifications for the choices of some of the parameters are given below. Since temperature elevations in response to heating may influence some of the properties used in the drug transport model, temperature dependence of these properties is also considered.

- 1. **Tissue related transport parameters**
- **Blood vessel surface area to tissue volume ratio**

The ratio of surface area of blood vessel to tissue volume has a direct influence on the amount of anticancer drug in the interstitial fluid. Its value depends strongly on the type of tissue and stage of tumor growth [37]. Pappenheimer et al. measured this in normal tissues [38], while Baxter and Jain [1] recommended using $70 {cm}^{-1}$ and $200 {cm}^{-1}$ for normal and tumor tissues, respectively. Grown tumors are mainly composed of three zones based on vascular density and cell proliferation; hypoxic zone, quiescent zone and proliferation. The hypoxic zone is formed mainly in the center of the tumor due to poor perfusion, in this area the vascular density is very poor. In the quiescent zone, angiogenesis is performed and perfusion is normal. But it has a lower vascular density than the proliferative zone. Accordingly, vascular density changes are defined as a quasi-sinusoidal function that is considered zero in the tumor center (Fig. S12).

Vascular damage under thermal ablation is considered, as with tissue damage based on the Arrhenius model (Eq.78).

- **Perfusion rate**

Spatial distribution of blood perfusion is considered like vascular density. On the other hand, blood perfusion rate ($\omega$) also depends on temperature [39] by the following relation:

| $\omega=\omega_{0}DS$ | (79) |
| --- | --- |

Where $\omega_{0}$ represents the time dependent perfusion at $37˚C$, and DS is the degree of vascular stasis with a value between 0 and 1 [40].

| $DS=e^{-\Omega\left( t \right)}$ | (80) |
| --- | --- |

- **Thermal conductivity**

Thermal conductivity like other parameters varies with temperature, which is defined as follows [41]:

| $k\left( t \right)=k_{0}+0.001161(T-T_{0})$ | (81) |
| --- | --- |

Where $k_{0}$ is the thermal conductivity at $T_{0}$, that $T_{0}$ is $37˚C$.

- **Bio-tissue thermal characteristics of tumor tissue**

The injection of MNPs results in the change of tumor region characteristics. Thus, the thermal characteristics of bio-tissue after injection can be determined by the properties of MNPs and tumor tissue, and also the volume fraction of MNPs. The values of density, specific heat ratio, and thermal conductivity are described in equations 82 to 85, respectively [42].

| $\rho=\rho_{0}\left( 1-\emptyset\right)+\emptyset\rho_{MNP}$ | (82) |
| --- | --- |
| $c=c_{0}\left( 1-\emptyset\right)+\emptyset c_{MNP}$ | (83) |
| $\frac{1}{k}=\frac{(1-\emptyset)}{k_{0}}+\frac{\emptyset}{k_{MNP}}$ | (84) |

Where $\rho_{0}$ and $c_{0}$ are the density and specific heat of tumor tissue before the MNPs are injected, respectively. $\rho_{MNP}$, $c_{MNP}$, and $k_{MNP}$ are the density, specific heat, and thermal conductivity of MNPs.

- **Absorption coefficient**

The absorption coefficient increases by the frequency enhancement and could be calculated by Eq. 85 [24]:

| $\alpha_{ABS}=\alpha_{0}\left( \frac{f}{f_{0}} \right)^{\beta}$ | (85) |
| --- | --- |

where $\beta$ is the absorption power, and $\alpha_{0}$ is the absorption coefficient at $f_{0} = 1 MHz$. The absorption coefficient changes linearly with the frequency for biological soft tissues [43], therefore, $\beta$ = 1.

- 1. **Drug related transport parameters**
     1. **Vascular permeability**

Vascular permeability coefficient measures the capacity of a microvessel wall to allow for the flow of substances in and out of the vasculature. The structure of microvessel wall and the molecular size of the transported substance are key determinants of permeability [44].

- **Free & bound drug**

Estimates of this parameter reported in the literature usually correspond to ‘effective permeability’, which is on the order of ${10}^{-7} m/s$ for albumin in both tumor and normal tissues. Compared with normal tissues, Gerlowski and Jain [45] found the vessel wall permeability to be 8 times higher in tumor tissues. Drug permeability variation based on temperature is logarithmic [9]. Hence, Zhan [9] estimated the fold increase in permeability for free and bound drug as follows:

| $\frac{P_{0}}{P}={10}^{0.7(T_{0}-T_{2})}$ | (86) |
| --- | --- |

Where $P_{0}$ and $P$ are the permeability of Free and bound drug at $T_{0}$ and $T$, respectively.

- **ThermoDox**

For a baseline temperature of 34 ˚C, extracellular concentrations of thermo sensitive liposome were found to have increased by 76-fold and 38-fold upon heating to 45˚C and 42˚C, respectively [46]. Based on these experimental data [46], a relationship between fold increase in permeability and temperature defined [9].

| $\frac{P}{P_{0}}=-10.54+5.76e^{\left( \frac{T-T_{0}}{5.44} \right)}+5.78e^{\left( \frac{T-T_{0}}{5.46} \right)}$ | (87) |
| --- | --- |

where $P_{0}$ and $P$ are the permeability of ThermoDox at $T_{0}$ and $T$, respectively.

- - 1. **Diffusion coefficient**

Diffusion coefficient is the constant of proportionality between the particle flux owing to particle diffusion and the gradient in the concentration of the species, which is also known as the driving force for diffusion.

- **Free & bound drug**

Diffusion coefficient is related to the molecular weight (MW) of the Dox [15], Temperature does not have a significant effect on the diffusion coefficient of Free and bound drug.

- **ThermoDox**

According to the Stokes-Einstein equation, diffusivity based on temperature and viscosity. So, the fold increase in diffusivity of ThermoDox as a function of temperature and viscosity are [9]:

| $\frac{D_{0}}{D}=\frac{T_{0}\mu}{T\mu_{0}}$ | (88) |
| --- | --- |

where $D_{0}$ and $D$ are the diffusivity coefficient ThermoDox at $T$ and $T_{0}$, respectively. Owing to the lack of relevant data, viscosity values are assumed to be those of water [47], whose dependence on temperature is given by Eq. 89 [9]. So, the fold increase in diffusivity of viscosity is given by Eq. 90 [9].

| $\mu_{w}=exp(5.1-0.03T+1.04\times{10}^{-4}T^{2})$ | (89) |
| --- | --- |
| $\frac{\mu}{\mu_{0}}=exp(-0.03\left( T-T_{0} \right)+1.04\times{10}^{-4}(T^{2}-T_{0}^{2}))$ | (90) |

- - 1. **Transmembrane rate**

The transmembrane parameter was determined by El-Kareh and Secomb [5] by curve fitting to data obtained from in vitro experiments [48]. It has been suggested that increased cellular uptake of Dox with heating will lead to the improved outcomes when the drug is administrated simultaneously with hyperthermia [5]. Based on data in [49], there is a 2.2-fold increase at 42˚C. Here, the fold increase at temperature $T$ is obtained by linear interpolation [9].

| $k_{tv}=0.24T-7.88$ | (91) |
| --- | --- |

- - 1. **Drug dose**

As a common anticancer drug, Dox is widely used in chemotherapy to treat various types of cancer, such as lymphoma, genitourinary, thyroid, and stomach cancer [50]. By interacting with DNA in cells, Dox can inhibit the process of DNA replication. Because of this mechanism of action, a high concentration of Dox in normal tissues can cause serious damage to healthy cells, known as side effects. In clinical therapy, the most serious toxicity is life-threatening cardiomyopathy [51], leading to heart failure. Side effects set a limit to the lifetime dose a patient can receive, which is approximately $550 mg$per unit body surface area [50]. The dose of Dox in clinical use is related to the patient’s body surface area. In each treatment cycle, the dose is between $50$ to $75 mg/m^{2}$ [50]. Body surface area (BSA) is determined from the following formula [52]

| $BSA=\left( \frac{Weight in kg}{70 kg} \right)^{0.73}\times(1.73m^{2})$ | (92) |
| --- | --- |

For a $70 kg$ patient, dosage of doxorubicin is in the range of $86.5\sim129.75$mg.

The initial concentration of encapsulated Dox is $0.0191 kg/m^{3}$, corresponding to a total dose of $50 mg/m^{2}$ in literature [53]. Higher doses can be used because Dox encapsulation reduces nonspecific distribution [5]. Therefore, higher concentrations can be considered for ThermoDox, which indicate a higher load of the drug ($2\times\left( 0.0191\frac{kg}{m^{3}} \right)$ and $3\times\left( 0.0191\frac{kg}{m^{3}} \right)$).

- - 1. **Drug release**

ThermoDox is designed to release its payload rapidly upon heating. The exact release rate varies according to the composition of liposome, its preparation procedure and heating temperature [54]. In order to be stable in the circulation for a longer period of time, the release rate must be very low. It has been reported that ThermoDox have good stability at body temperature with a release rate of $3\times{10}^{-4}(s^{-1})$ [55]. A ThermoDox will be efficient when it discharges its payload quickly at high temperatures. It has been reported that ThermoDox is able to release its payload explosively at a temperature of $43 ℃$ with a release rate of $0.3(s^{-1})$ [56]. It does not have to be a local temperature of $41.3 ^{\circ} C$ for ThermoDox to release its payload effectively, they are able to release their payload in less than one minute within their melting temperature range ($39-40 ^{\circ} C$) [57]. According to the literature, in this study, the release rate based on temperature is considered in Table S6 and for temperatures above $41.3 ^{\circ} C$, the release rate is assumed to be $0.3(s^{-1})$.

1. **Solution strategy**

This model is based on a spherical tumor that has been simulated in 2D axisymmetric. A tumor with a radius of 10 mm is located in the center of a normal tissue that is about three times the size of the tumor. Fig. S13 shows a schematic of the tumor and boundary conditions. Fig. S13I shows a schematic of the solution domain and boundary conditions. Because MNPs accumulate next to each other after entering the interstitium, they are considered as clusters. Nearly 2000 clusters with a diameter of 100 μm in the proliferation zone are uniformly distributed, which is the heat source under the magnetic field in the tumor. The size of MNPs is considered to be more than 12 nm, so because they are larger than the pore size of normal tissue vessels (5-6 nm), the distribution of MNPs in normal tissue is not considered (Fig. S13II). A single-element transducer with a specific physical property (Table S7) has been used to ablation the tumor. The location of the transducer is defined so that its focal point is at the center of the tumor (Fig. S13III). The simulation was performed using the commercial finite element software COMSOL Multiphysics 5.5 (COMSOL, Inc., Burlington, MA, USA). Boundary conditions between tumor and normal tissue are applied automatically and the remaining boundary conditions are defined by the user for the software. Solution field meshing is considered in the first and second study of triangular elements with a finer level, but for the third case where acoustics was performed, a maximum element size $1483[m/s]/f/5$ was considered.

| Table S1. The spatial-mean temperature $(℃)$ of tissue and blood variations under different magnetic field conditions and various MNP size. | | | | | |
| --- | --- | --- | --- | --- | --- |
| Strength $(kA\cdot m^{-1})$- Frequency$(kHz)$ | Physics | MNPs Size | | | |
|  |  | 12 | 15 | 18 | 21 |
| 7-300 | Tumor tissue | 37.037 | 37.247 | 38.893 | 55.348 |
|  | Normal tissue | 37.007 | 37.042 | 37.120 | 37.787 |
|  | Tumor blood | 37.011 | 37.071 | 37.547 | 42.752 |
|  | Tbn | 37.005 | 37.033 | 37.153 | 37.198 |
| 9-300 | Tumor tissue | 37.062 | 37.380 | 39.726 | 59.591 |
|  | Normal tissue | 37.011 | 37.064 | 37.146 | 37.864 |
|  | Tumor blood | 37.018 | 37.110 | 37.787 | 44.895 |
|  | Normal blood | 37.008 | 37.051 | 37.124 | 37.426 |
| 11-300 | Tumor tissue | 37.090 | 37.525 | 40.571 | 65.237 |
|  | Normal tissue | 37.015 | 37.089 | 37.171 | 38.083 |
|  | Tumor blood | 37.026 | 37.152 | 38.032 | 45.611 |
|  | Normal blood | 37.012 | 37.071 | 37.156 | 37.665 |
| 13-300 | Tumor tissue | 37.122 | 37.678 | 41.750 | 71.083 |
|  | Normal tissue | 37.021 | 37.095 | 37.340 | 38.405 |
|  | Tumor blood | 37.035 | 37.196 | 39.095 | 49.150 |
|  | Normal blood | 37.016 | 37.091 | 37.316 | 37.873 |
| 15-300 | Tumor tissue | 37.157 | 37.864 | 42.905 | 78.868 |
|  | Normal tissue | 37.027 | 37.092 | 37.477 | 39.566 |
|  | Tumor blood | 37.045 | 37.241 | 39.449 | 51.261 |
|  | Normal blood | 37.021 | 37.089 | 37.418 | 38.079 |

| Table S2. Physiological parameters. | | | | | | | |
| --- | --- | --- | --- | --- | --- | --- | --- |
| Parameter | Definition | Unit | Tumor Tissue | Normal Tissue | Blood | Whole  Body | Ref. |
| $S/V$ | Surface area of blood vessels per unit tissue volume | $m^{-1}$ | 20000 | 7000 | - | - | [1, 2] |
| $K_{v}$ | Hydraulic conductivity of the micro-vascular wall | $m/Pa\cdot s$ | $2.1\times{10}^{-11}$ | $2.7\times{10}^{-12}$ | - | - | [1, 2] |
| $\rho$ | Tissue density | $kg/m^{3}$ | 1000 | 1000 | 1060 | - | [31, 58] |
| $\mu$ | Dynamic viscosity of interstitial fluid | $kg/m\cdot s$ | $7.8\times{10}^{-4}$ | $7.8\times{10}^{-4}$ | - | - | [52] |
| $\frac{1}{\kappa}$ | Permeability of the interstitial space | $m^{-2}$ | $4.56\times{10}^{16}$ | $2.21\times{10}^{17}$ | - | - | [1, 52] |
| $p_{v}$ | Vascular fluid pressure | $Pa$ | 2080 | 2080 | - | - | [1, 10, 11] |
| $\pi_{v}$ | Osmotic pressure of the plasma | $Pa$ | 2666 | 2666 | - | - | [1, 10, 11] |
| $\pi_{i}$ | Osmotic pressure of interstitial fluid | $Pa$ | 2000 | 1333 | - | - | [1, 10, 11] |
| $\sigma_{T}$ | Average osmotic reflection coefficient | 1 | 0.82 | 0.91 | - | - | [1, 10, 11] |
| $K_{ly}S_{ly}/V$ | Hydraulic conductivity of the lymphatic wall times surface area of lymphatic vessels per unit volume of tumor tissue | $\frac{1}{(pa\cdot s)}$ | 0 | $4.17\times{10}^{-7}$ | - | - | [52] |
| $p_{ly}$ | Intra-lymphatic pressure | $Pa$ | 0 | 0 | - | - | [52] |
| $D_{c}$ | Cell density | ${10}^{5}cell/m^{3}$ | ${10}^{10}$ | ${10}^{10}$ | - | - | [6, 9] |
| $c_{0}$ | Ultrasound speed | $m/s$ | 1550 | 1550 | 1540 | - | [31] |
| $c$ | Specific heat | $J/(kg\cdot K)$ | 3800 | 3600 | 3770 | - | [31] |
| $k$ | Thermal conductivity | $W/(m\cdot K)$ | 0.552 | 0.512 | 0.53 | - | [31] |
| $w_{b0}$ | Blood perfusion rate at 37 $℃$ | $s^{-1}$ | 0.002 | 0.018 | - | - | [59] |
| $R_{g}$ | Universal gas constant | $J/(mol\cdot K)$ | 8.314 | 8.314 | - | - | [39] |
| $\Delta E$ | Activation energy | $J/mol$ | $6.67\times{10}^{5}$ | $6.67\times{10}^{5}$ | - | - | [39] |
| $A_{f}$ | Frequency factor | $s^{-1}$ | $1.98\times{10}^{106}$ | $1.98\times{10}^{106}$ | - | - | [39] |
| $V_{BB}$ | Total blood volume in body | l | - | - | - | 5.53 | [60] |
| $H_{ct}$ | Hematocrit | 1 | 0.19 | 0.45^*^ | - | 0.45 | [61, 62] |
| $V_{TV}$ | Volume fraction of tissue vascular space | 1 | 0.092 | 0.0322^*^ | - | - | [63] |
| $V_{BT}$ | Volume of body tissue | l | - | - | - | 64.47 | [60] |
| $Q_{m}$ | The heat generated by metabolism | $\frac{W}{m^{-3}}$ | 0 | 0 | - | - | [16, 40] |
| * The related values are assumed in this study. | | | | | | | |

| Table S3. Parameters for doxorubicin | | | | | |
| --- | --- | --- | --- | --- | --- |
| Parameter | Definition | Unit | Free Doxorubicin | Bound Doxorubicin | Ref. |
| $P_{tumor}$ | Permeability of vasculature wall in tumor tissue | $m/s$ | $3.6\times{10}^{-6}$ | $7.8\times{10}^{-9}$ | [52, 64] |
| $P_{\mathrm{normal}}$ | Permeability of vasculature wall in normal tissue | $m/s$ | $3.75\times{10}^{-7}$ | $2.5\times{10}^{-9}$ | [52, 64] |
| $D_{tumor}$ | Diffusion coefficient in interstitial fluid of tumor | $m^{2}/s$ | $3.4\times{10}^{-10}$ | $8.89\times{10}^{-12}$ | [52, 65-67] |
| $D_{\mathrm{normal}}$ | Diffusion coefficient in interstitial fluid of normal | $m^{2}/s$ | $1.58\times{10}^{-10}$ | $4.17\times{10}^{-12}$ | [52, 65-67] |
| $\sigma_{d}$ | Osmotic reflection coefficient | 1 | 0.15 | 0.82 | [68] |
| $k_{a}$ | Doxorubicin-protein binding rate | $s^{-1}$ | 0.833 | - | [6] |
| $k_{d}$ | Doxorubicin-protein dissociation rate | $s^{-1}$ | - | 0.278 | [6] |
| $\varphi$ | Tumor fraction extracellular space | 1 | 0.4 | - | [6] |
| $V_{max}$ | Rate of trans-membrane transport | $kg/{10}^{5}cell\cdot s$ | $4.67\times{10}^{-15}$ | - | [6, 48] |
| $k_{e}$ | Michaelis constant for transmembrane transport | $kg/m^{3}$ | $2.19\times{10}^{-4}$ | - | [6, 48] |
| $k_{i}$ | Michaelis constant for transmembrane transport | $kg/{10}^{5}cells$ | $1.37\times{10}^{-12}$ | - | [6, 48] |
| $f_{max}$ | Cell-kill rate constant | $s^{-1}$ | $1.67\times{10}^{-5}$ | - | [14] |
| ${EC}_{50}$ | Drug concentration producing 50% of $f_{max}$ | $kg/{10}^{5}cells$ | $5\times{10}^{-13}$ | - | [14] |
| $k_{c}$ | Cell proliferation rate | $s^{-1}$ | $3\times{10}^{-6}$ | - | [69] |
| $k_{g}$ | Cell physiologic degradation rate | $s^{-1}$ | $3\times{10}^{-16}$ | - | [69] |
| A | Parameter for pharmacokinetic model | $m^{-1}$ | 130 | - | [6, 7] |
| ${CL}_{Tumor}$ | Plasma clearance in tissue | $s^{-1}$ | $2.43\times{10}^{-3}$ | 0 | [70, 71] |
| ${CL}_{Sys}$ | Plasma clearance in Systemic | $s^{-1}$ | $1.1 \times{10}^{-3}$ | 0 | [72] |
| $k_{P}$ | Transfer constant free drug from systemic plasma to tissue | $s^{-1}$ | $1.6 \times{10}^{-3}$ | $9.6 \times{10}^{-5}$^*^ | [72] |
| $k_{t}$ | Transfer constant free drug from tissue to systemic plasma | $s^{-1}$ | $4.8 \times{10}^{-5}$ | $2.8 \times{10}^{-7}$^*^ | [72] |
| $t_{\frac{1}{2}}^{\alpha}$ | Half-life of doxorubicin in plasma | min | 4.75 | - | [7] |
| * The related values are assumed in this study. | | | | | |

| Table S4. Parameters for liposome | | | | | | |
| --- | --- | --- | --- | --- | --- | --- |
| Parameter | Definition | Unit | Tumor Tissue | Normal Tissue | Systemic | Ref. |
| $P_{L}$ | Liposome permeability of vasculature wall | $m/s$ | $5.44\times{10}^{-12}$ | $0$^*^ | - | [73] |
| $D_{L}$ | Hydraulic conductivity of the micro-vascular wall | $m^{2}/s$ | $2.2\times{10}^{-12}$ | $1.41\times{10}^{-12}$ | - | [73] |
| $\sigma_{L}$ | Reflection coefficient for liposome | 1 | 95 | 1 | - | [40] |
| ${CL}_{Lipt}$ | TSL clearance in tissue plasma | $s^{-1}$ | $2.228\times{10}^{-4}$ | $2.228\times{10}^{-4}$ | - | [53] |
| ${CL}_{Lips}$ | TSL clearance in systemic plasma | $s^{-1}$ | - | - | $9.417\times{10}^{-6}$ | [60] |
| * The related values are assumed in this study. | | | | | | |

| Table S5. Material parameters of the MNPs $(\gamma-{Fe}_{2}O_{3})$ | | | | |
| --- | --- | --- | --- | --- |
| Parameter | Definition | Unit | Value | Ref. |
| $\mu_{0}$ | Permeability of free space | $H/m$ | $4\pi\times{10}^{-7}$ | [74-76] |
| $M_{d}$ | Domain magnetization | $kA/m$ | 414 | [74-76] |
| $k_{B}$ | Boltzmann constant | $J/K$ | ${1.38\times10}^{-23}$ | [74-76] |
| $\eta$ | Dynamic viscosity | $kg/m\cdot s$ | $0.00235$ | [74-76] |
| $\tau_{0}$ | Time constant | $s$ | ${10}^{-9}$ | [74-76] |
| $k_{a}$ | Anisotropy constant | $kJ/m^{3}$ | $4.7$ | [74-76] |
| $\delta$ | Surfactant layer thickness | $nm$ | $2$ | [74-76] |
| $\rho$ | Density | $kg/m^{3}$ | $4600$ | [74-76] |
| $c$ | Specific heat | $J/kg\cdot K$ | $746$ | [74-76] |
| $k$ | Thermal conductivity | $W/m\cdot K$ | $528$ | [74-76] |

| Table S6. Release rates at various temperatures | | | | | |
| --- | --- | --- | --- | --- | --- |
| $T (℃)$ | 37 | 38 | 39 | 40 | 41.3 |
| $k_{p}$ | 0.0003 | 0.0047 | 0.142 | 0.221 | 0.3 |

| Table S7. HIFU transducer parameters | | | |
| --- | --- | --- | --- |
| Parameter | Inside diameter | Outside diameter | Focal length |
| Unit | mm | mm | mm |
| Value | 20.0 | 70.0 | 62.64 |

| 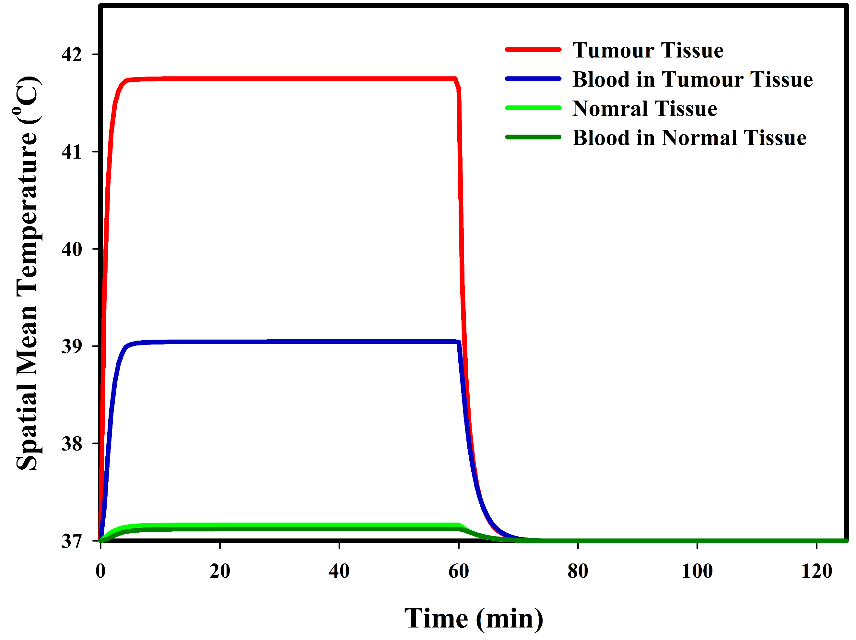 | At 30 min | |
| --- | --- | --- |
|  | 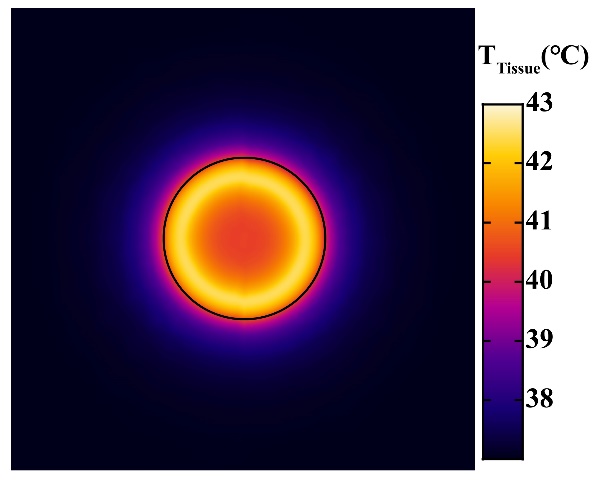 | 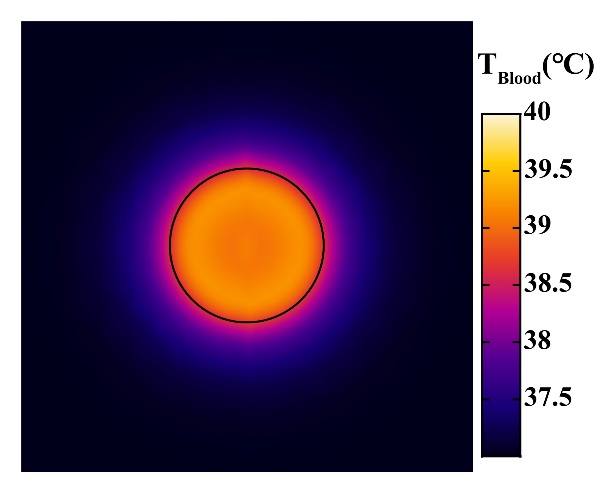 |
|  | At 63 min | |
|  | 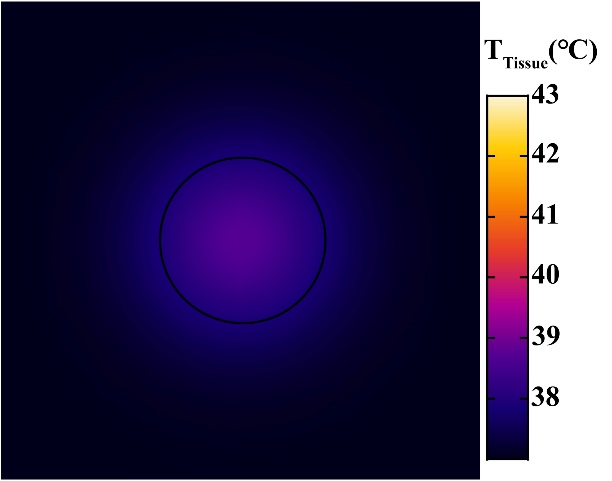 | 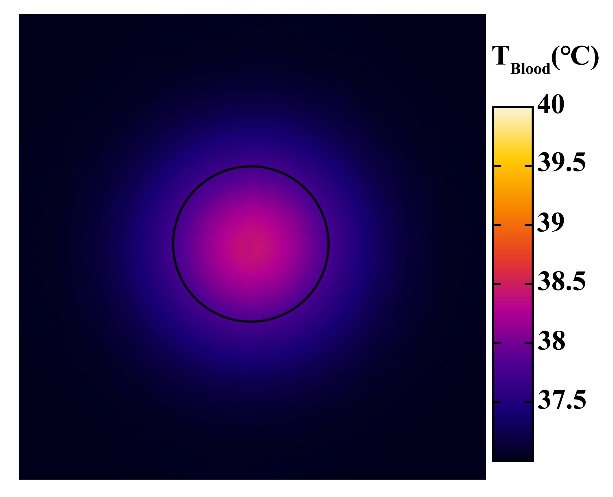 |
| (A) | (B) | |
| Fig. S1. Temperature distribution in tissues and blood vessels; Maximum temperature occurs in the proliferation zone of tumor tissue where MNPs accumulate. After 10 minutes of applying the magnetic field, the temperature reaches its maximum value. Normal tissue is not significantly affected by tumor tissue temperature due to high perfusion. Its maximum temperature is less than $39 ℃$, which is not biologically damaged. Blood temperature is lower than tissue temperature due to circulating. Because the perfusion is weak in the center of the tumor tissue, this area has a higher temperature after the magnetic field is exposed (at 65min). It also takes about 20 min for the tissue temperature to return to normal. | | |

| **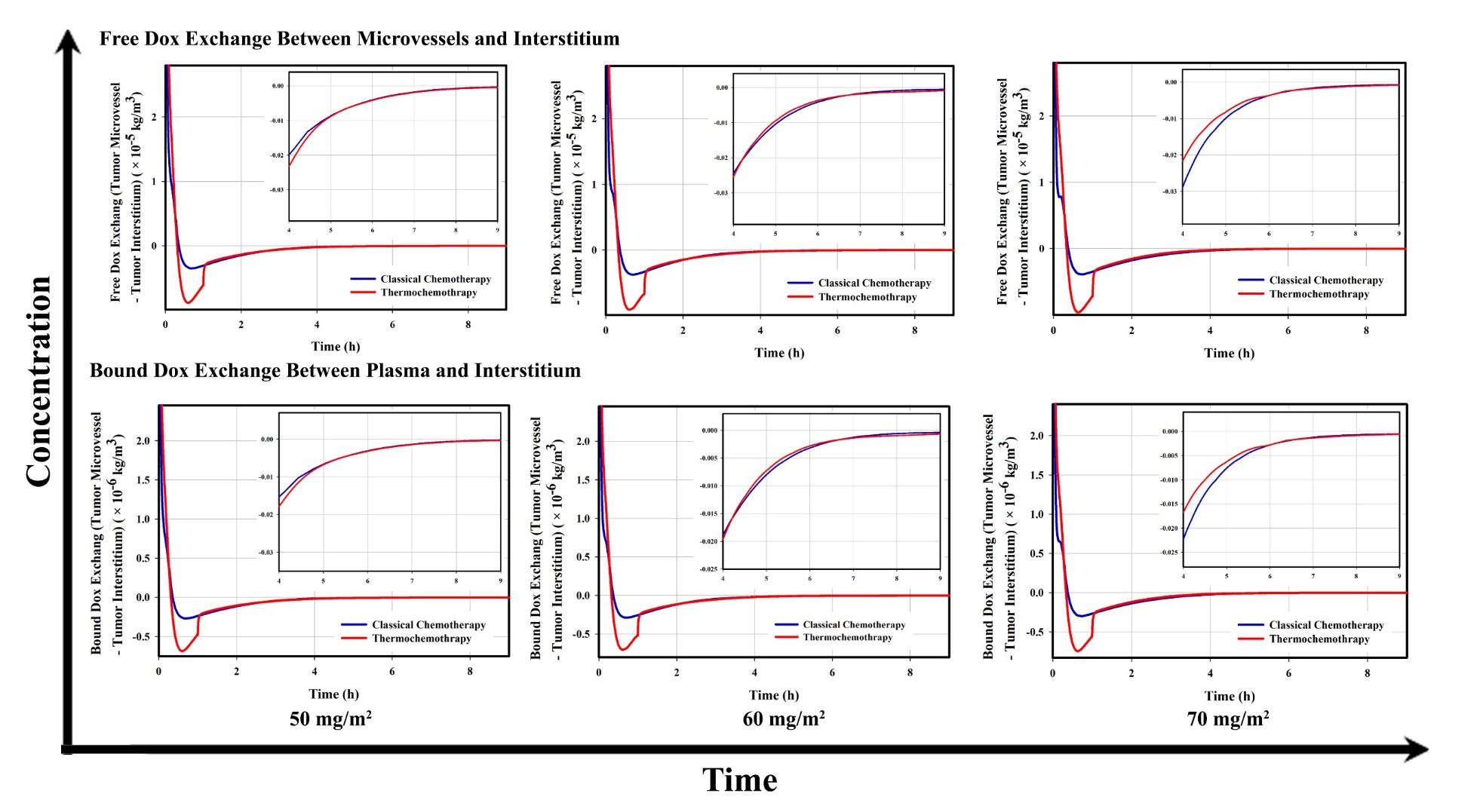** |
| --- |
| Fig. S2. Free- and bound drug exchange between blood and tumor interstitium; In the early stages, the free- and bound drug enters the interstitium from the vessels, after the free- and bound drug concentration in the tumor interstitium is much higher than the vessels, the free- and bound enters the bloodstream from the interstitium. Mild hypothermia increases the size of the pores in the walls of the arteries, which increases free- and bound drug exchange. It is clear that the rate of free drug exchange is the same for the three doses because the transvascular capacity is constant and independent of the concentration. The only difference is related to its maximum (${Max}_{50 \left( \frac{mg}{m^{2}} \right)}=13.976 \left( \times\frac{{10}^{-5}kg}{m^{3}} \right), {Max}_{60 \left( \frac{mg}{m^{2}} \right)}=16.772 (\times\frac{{10}^{-5}kg}{m^{3}}),{Max}_{70 \left( \frac{mg}{m^{2}} \right)}=19.567 (\times\frac{{10}^{-5}kg}{m^{3}})$). This is also true for bound drugs (${Max}_{50 \left( \frac{mg}{m^{2}} \right)}=11.09 \left( \times\frac{{10}^{-7}kg}{m^{3}} \right)$,${Max}_{60 \left( \frac{mg}{m^{2}} \right)}= 13.3080 \left( \times\frac{{10}^{-7}kg}{m^{3}} \right)$, ${Max}_{70 \left( \frac{mg}{m^{2}} \right)}=15.527(\times\frac{{10}^{-7}kg}{m^{3}})$). |

| **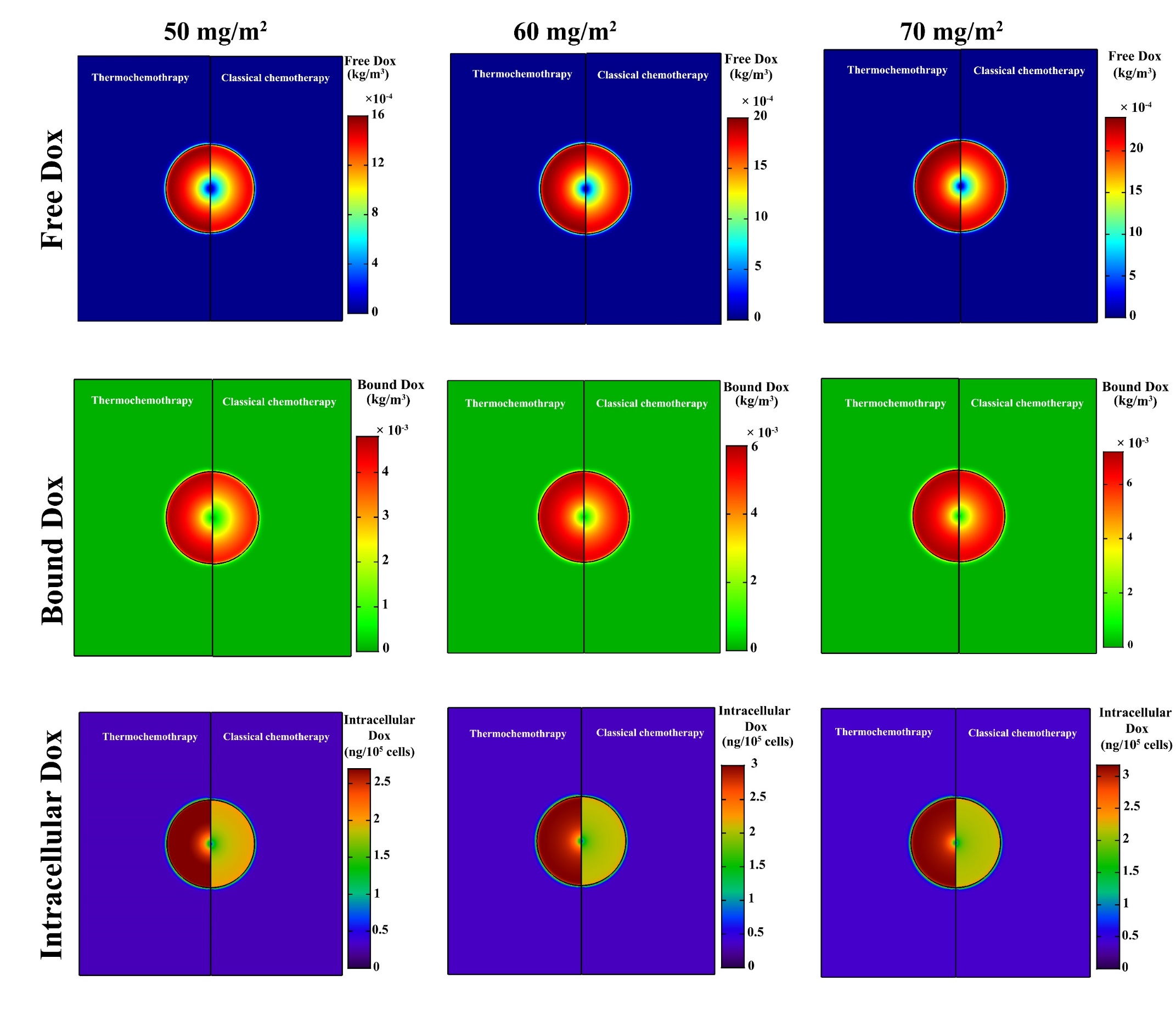** |
| --- |
| Fig. S3. Spatial distribution of the drug at its maximum amount; The maximum amount of free- and bound drug is accumulated in the proliferation zone and the hypoxia zone has the lowest amount of free- and bound drug accumulated due to the important resistance due to high IFP and poor vascular density in this zone. Due to the delay in transmembrane and deeper penetration of the free drug (with lower orders), cells in deeper regions are also able to internalize the drug to an acceptable degree. Normal tissue accumulates less drug in the interstitium due to poor vascular permeability, although more drug accumulates at the boundary with tumor tissue due to exchange between the two tissues. |

| **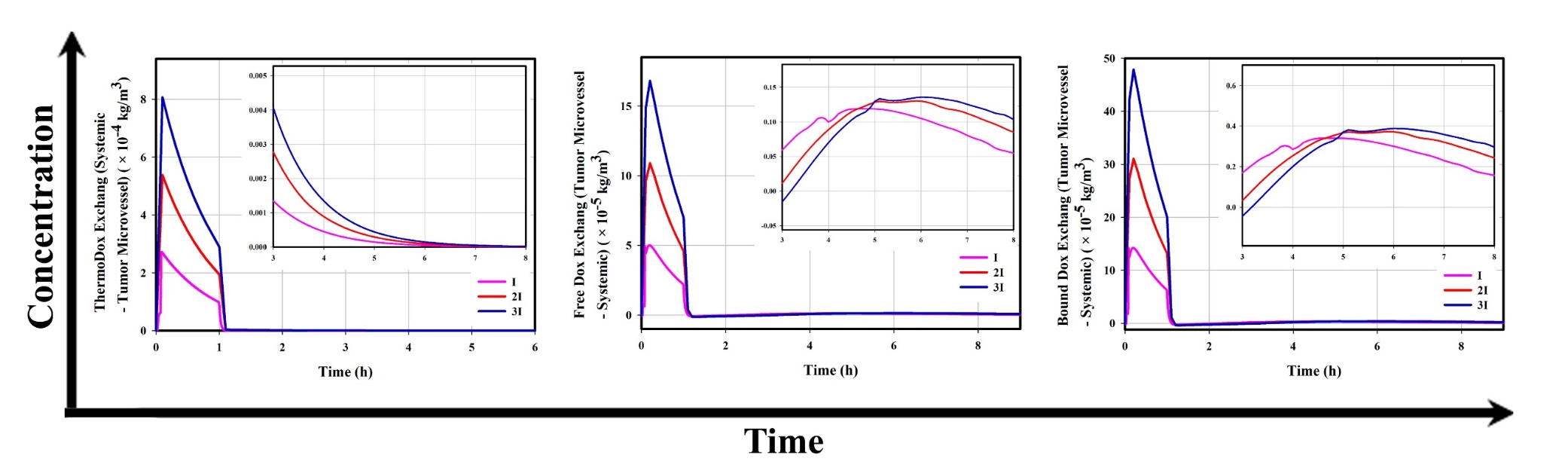** |
| --- |
| Fig. S4. The exchange of therapeutic agents between systemic plasma and tumor plasma; Decreased ThermoDox levels in the capillary network of the tumor due to extravasation and drug release have resulted in almost one-way exchange from systemic plasma to plasma tumor. While high concentrations of free- and bound drug in tumor plasma cause drug exchange from plasma tumor to systemic plasma. The determining factor in the rate of exchange is perfusion and the ratio of tumor plasma volume and systemic plasma volume ($I=0.0191\frac{kg}{m^{3}}, 2I=0.0382 \frac{kg}{m^{3}}, 3I=0.0573 \frac{kg}{m^{3}}$). |

| **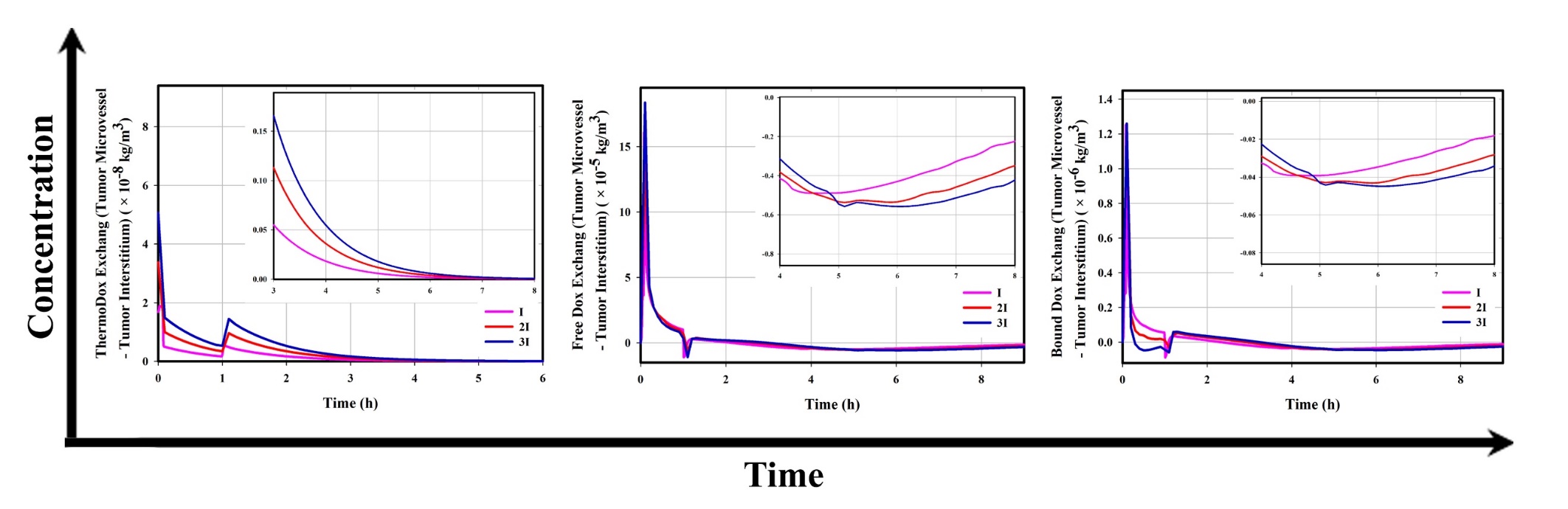** |
| --- |
| Fig. S5. The exchange of therapeutic agents between tumor plasma and tumor interstitium; ThermoDox continuously enters the tumor interstitium from the tumor plasma through the vascular pores, which is influenced by vascular permeability. Since vascular permeability is limited for particles about 100 nm in size, transvascular is very weak. The free- and bound drug enters the tumor interstitium quickly from the vessel in the first moments, after one hour when the drug release stops, at the moment the drug concentration in the tissue, it exceeds the vessel, so part of the drug returns to the vessel. Because the bound drug is not absorbed by the cancer cell, the concentration in the tissue increases and they return to the vessel sooner ($I=0.0191\frac{kg}{m^{3}}$, $2I=0.0382 \frac{kg}{m^{3}}$,$3I=0.0573 \frac{kg}{m^{3}}$). |

| **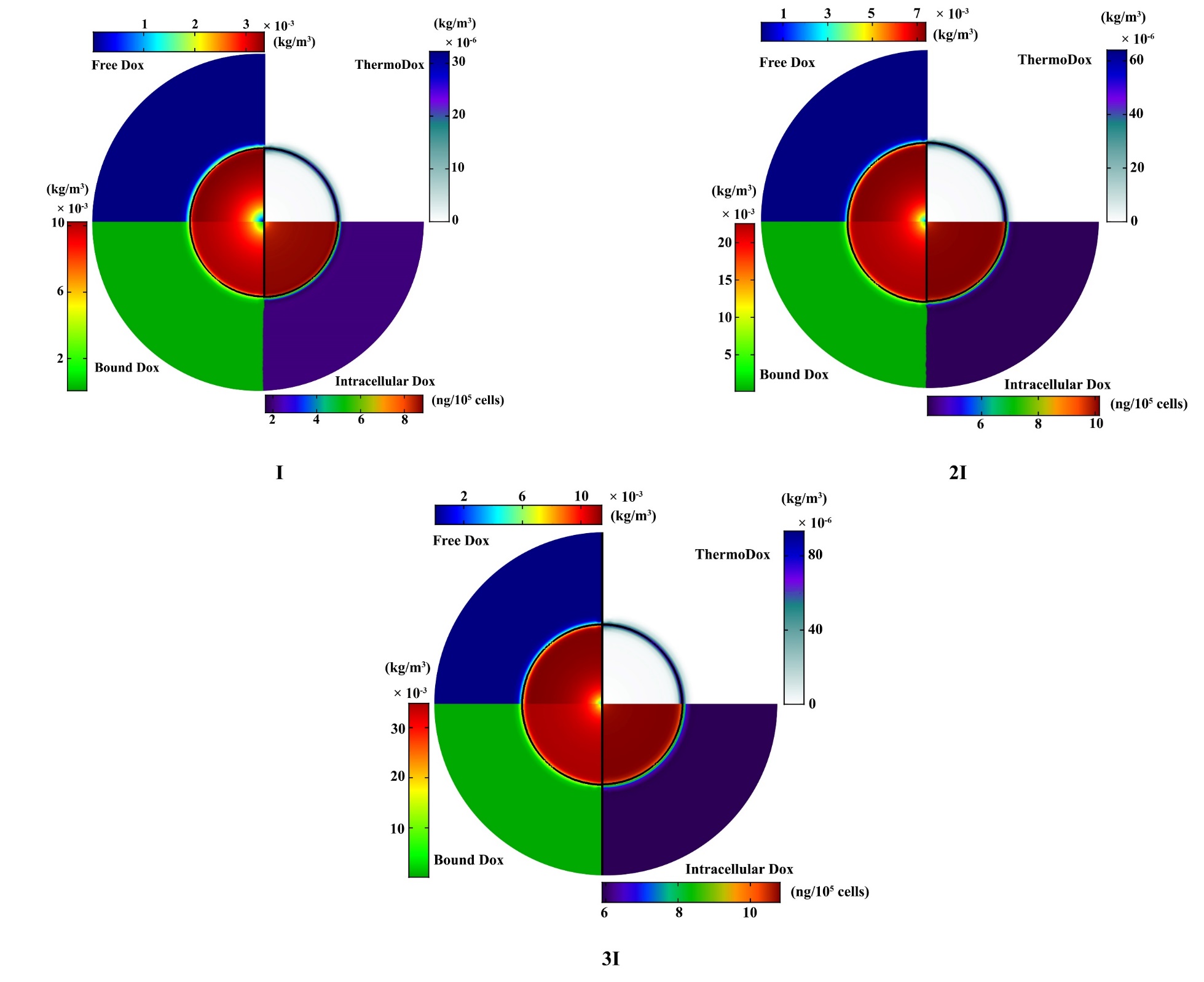** |
| --- |
| Fig. S6. Spatial distribution of the therapeutic agent at their maximum amount; ThermoDox accumulates at the tumor boundary due to poor diffusion. Free- and bound drugs are able to reach deeper areas of the tumor due to the high concentration gradient and reliance on the diffusion mechanism. This causes the drug to infect almost all tumor cells. |
| **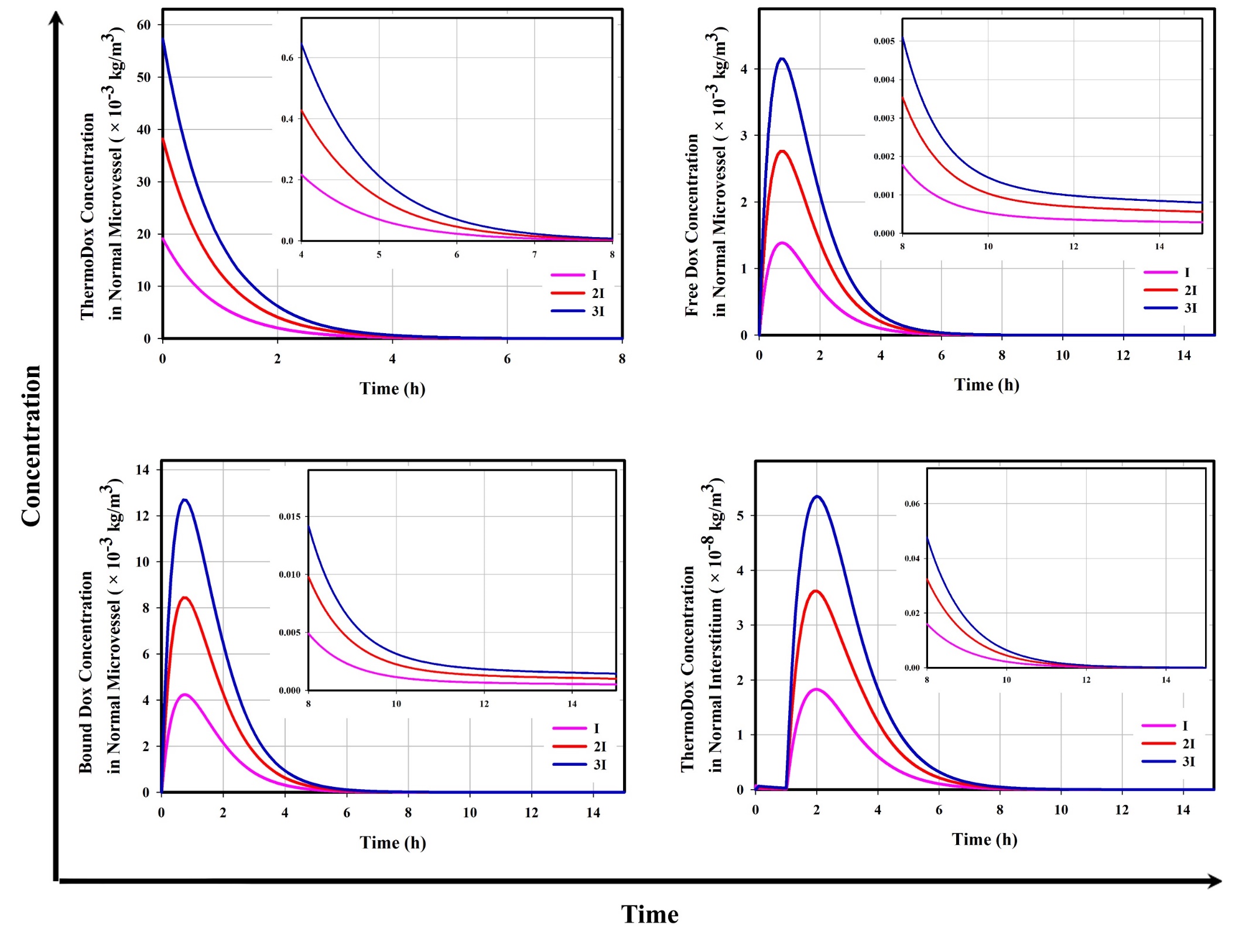** |
| Fig. S7. Temporal distribution therapeutic agents in normal tissue plasma and interstitium; Low heat and poor permeability of normal tissue vessels have caused the concentration of therapeutic agents in the capillary network of normal tissue is not significantly different from systemic plasma. ThermoDox in normal tissue has a very low concentration due to exchange with tumor tissue |

| **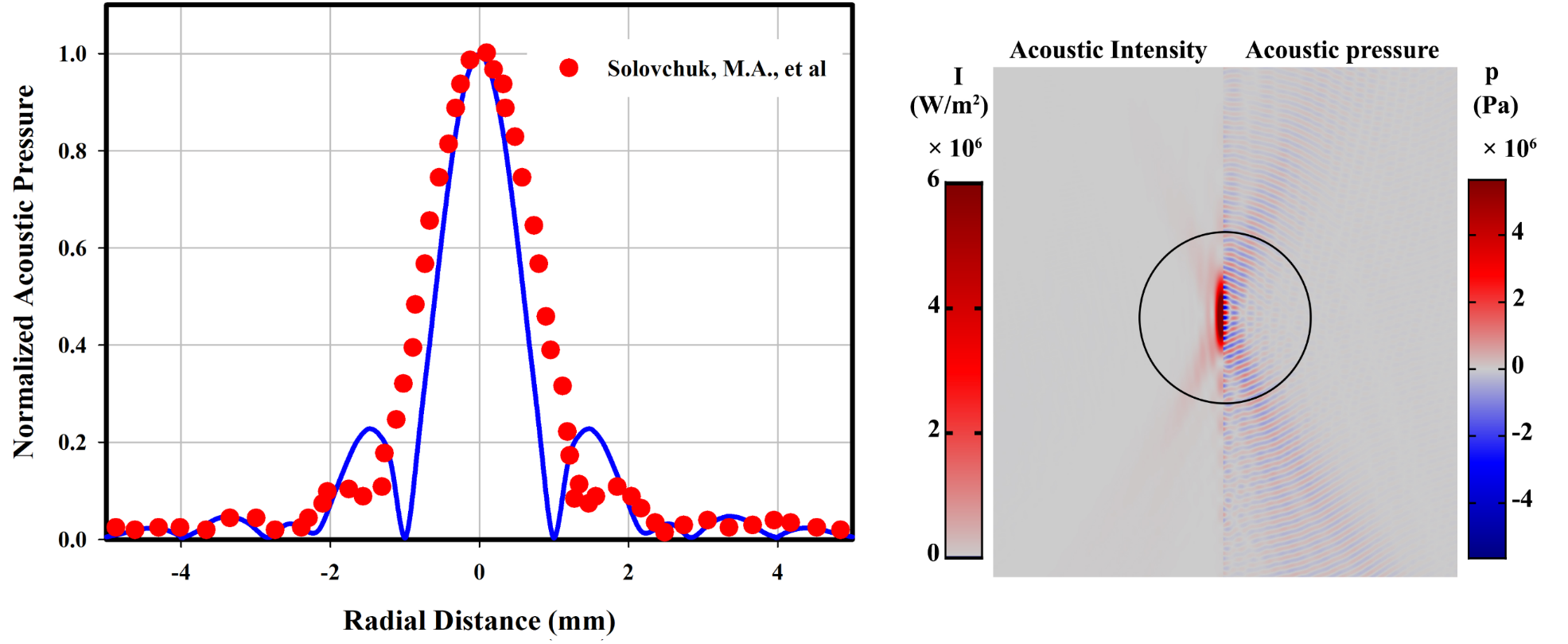** |
| --- |
| Fig. S8. Distribution of acoustic pressure and acoustic intensity at the focal point. For acoustic validation, the radial variations of acoustic pressure are studied with Solovchuk, M.A et al. [30] experimental results. |

| 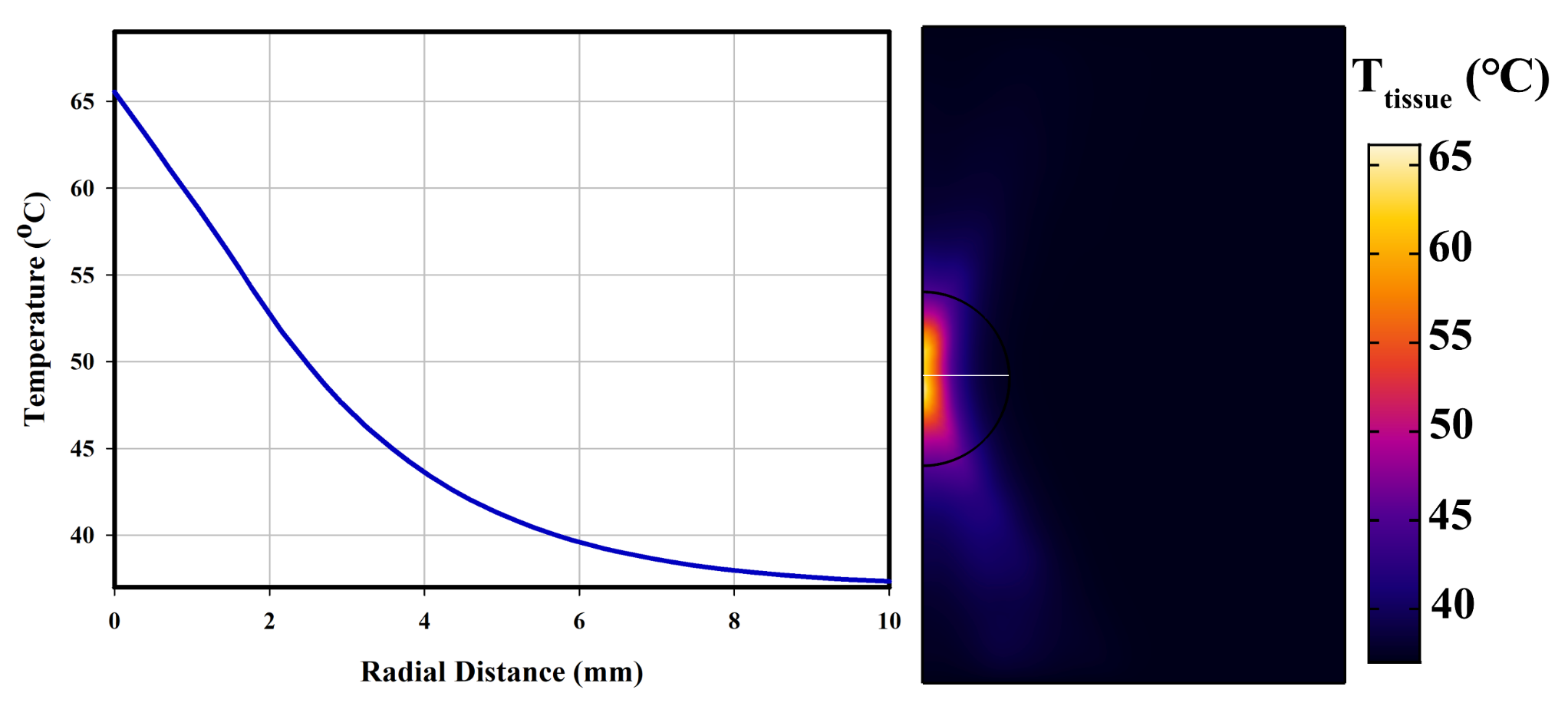 |
| --- |
| Fig. S9. Spatial distribution of temperature under HIFU, The maximum temperature occurs at the focal point. The high-temperature zone extends along the axis. |

| **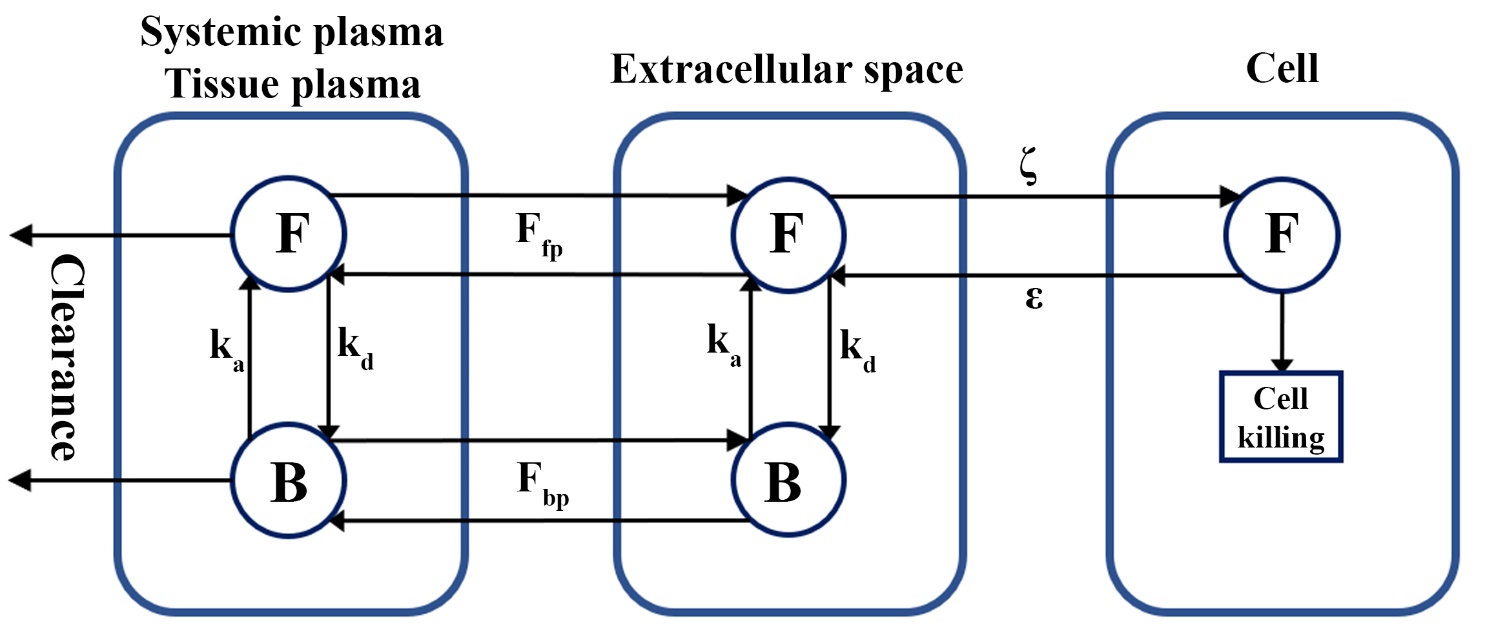** |
| --- |
| Fig. S10. Schematic diagram of multiple compartments used in classical chemotherapy (F: Free drug, B: Bound drug). |

| 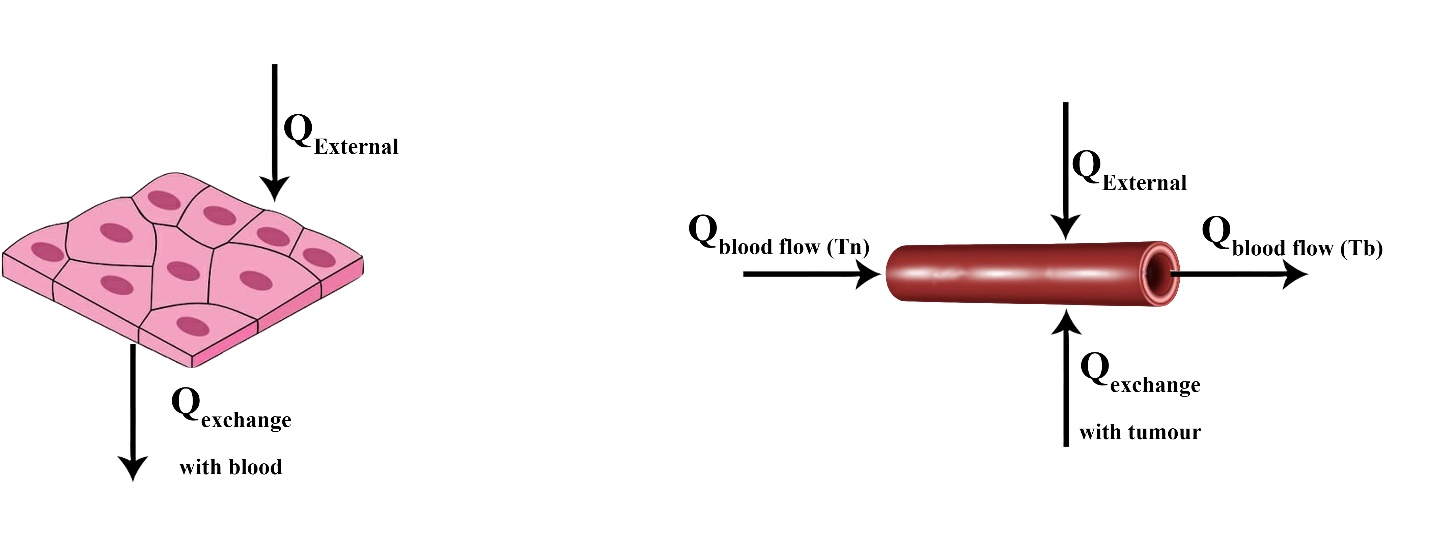 |
| --- |
| Fig. S11. Schematic representation of heat transfer under external stimuli tissue and blood. |

| 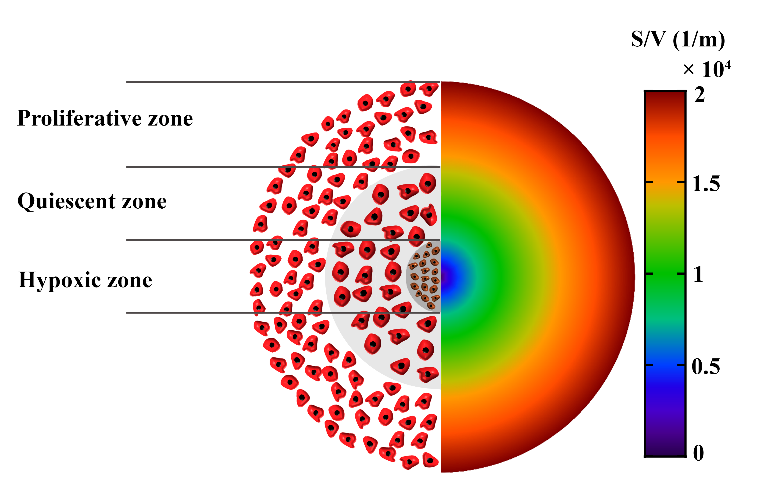 |
| --- |
| Fig. S12. vascular density variations in tumor tissue |

| 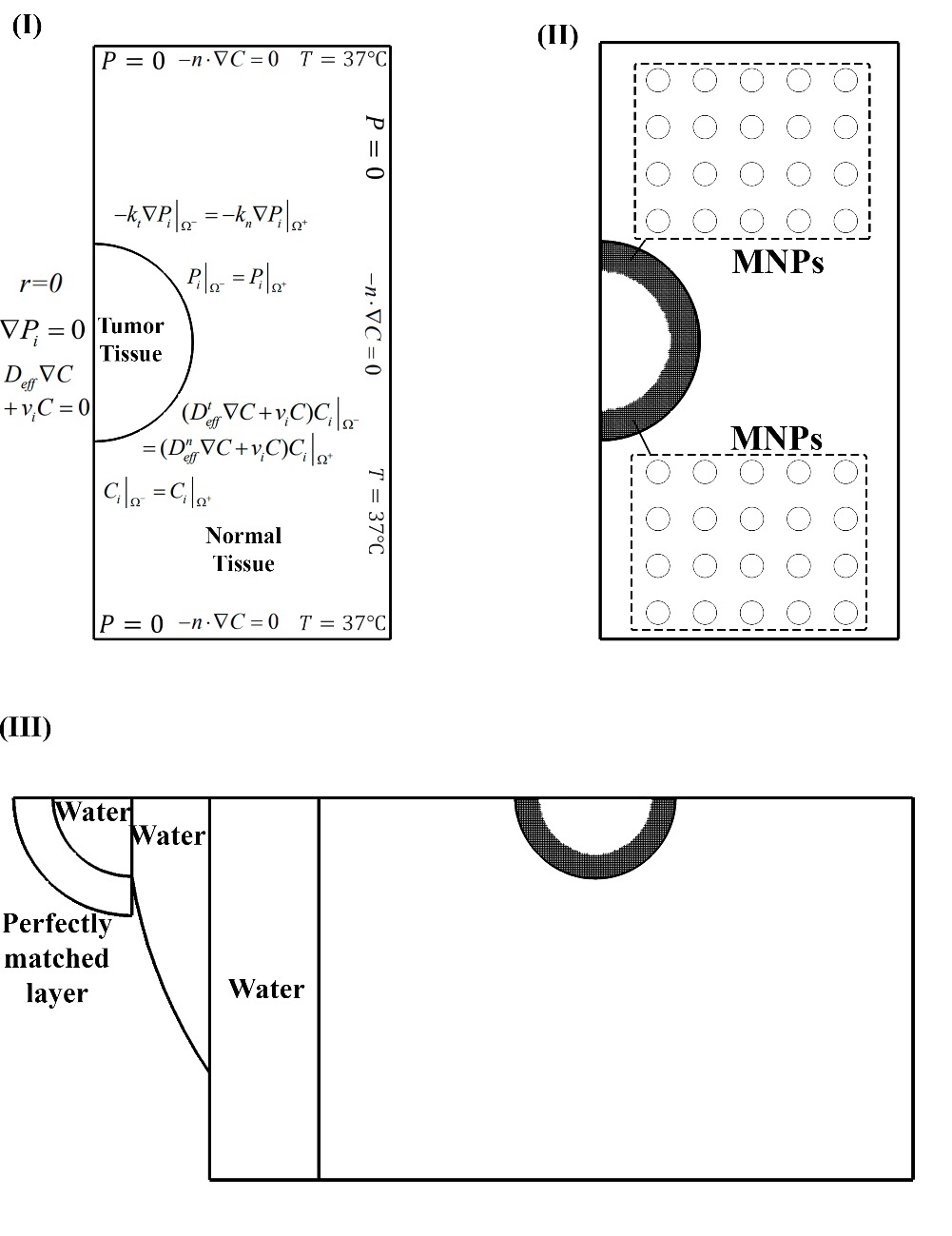 |
| --- |
| Fig. S13. Computational domain and boundary conditions employed. |

**Reference**

[1] L.T. Baxter, R.K. Jain, Transport of fluid and macromolecules in tumors. I. Role of interstitial pressure and convection, Microvascular research, 37 (1989) 77-104.

[2] M. Soltani, P. Chen, Numerical modeling of fluid flow in solid tumors, PloS one, 6 (2011) e20344.

[3] F.M. Kashkooli, M. Soltani, M. Rezaeian, E. Taatizadeh, M.-H. Hamedi, Image-based spatio-temporal model of drug delivery in a heterogeneous vasculature of a solid tumor—Computational approach, Microvascular research, 123 (2019) 111-124.

[4] W.M. Saltzman, Drug delivery: engineering principles for drug therapy, Oxford University Press2001.

[5] A.W. El-Kareh, T.W. Secomb, A mathematical model for comparison of bolus injection, continuous infusion, and liposomal delivery of doxorubicin to tumor cells, Neoplasia (New York, NY), 2 (2000) 325.

[6] S. Eikenberry, A tumor cord model for doxorubicin delivery and dose optimization in solid tumors, Theoretical Biology and Medical Modelling, 6 (2009) 16.

[7] J. Robert, A. Illiadis, B. Hoerni, J.-P. Cano, M. Durand, C. Lagarde, Pharmacokinetics of adriamycin in patients with breast cancer: correlation between pharmacokinetic parameters and clinical short-term response, European Journal of Cancer and Clinical Oncology, 18 (1982) 739-745.

[8] R.F. Greene, J.M. Collins, J.F. Jenkins, J.L. Speyer, C.E. Myers, Plasma pharmacokinetics of adriamycin and adriamycinol: implications for the design of in vitro experiments and treatment protocols, Cancer research, 43 (1983) 3417-3421.

[9] W. Zhan, Mathematical modelling of drug delivery to solid tumour, (2014).

[10] L.T. Baxter, R.K. Jain, Transport of fluid and macromolecules in tumors. II. Role of heterogeneous perfusion and lymphatics, Microvascular research, 40 (1990) 246-263.

[11] L.T. Baxter, R.K. Jain, Transport of fluid and macromolecules in tumors. IV. A microscopic model of the perivascular distribution, Microvascular research, 41 (1991) 252-272.

[12] A. Soundararajan, A. Bao, W.T. Phillips, R. Perez III, B.A. Goins, [186Re] Liposomal doxorubicin (Doxil): in vitro stability, pharmacokinetics, imaging and biodistribution in a head and neck squamous cell carcinoma xenograft model, Nuclear medicine and biology, 36 (2009) 515-524.

[13] Y. Huang, B. Gu, C. Liu, J. Stebbing, W. Gedroyc, M. Thanou, X.Y. Xu, Thermosensitive Liposome-Mediated Drug Delivery in Chemotherapy: Mathematical Modelling for Spatio–temporal Drug Distribution and Model-Based Optimisation, Pharmaceutics, 11 (2019) 637.

[14] R.E. Eliaz, S. Nir, C. Marty, F.C. Szoka, Determination and modeling of kinetics of cancer cell killing by doxorubicin and doxorubicin encapsulated in targeted liposomes, Cancer research, 64 (2004) 711-718.

[15] H.H. Pennes, Analysis of tissue and arterial blood temperatures in the resting human forearm, Journal of applied physiology, 1 (1948) 93-122.

[16] M. Rezaeian, A. Sedaghatkish, M. Soltani, Numerical modeling of high-intensity focused ultrasound-mediated intraperitoneal delivery of thermosensitive liposomal doxorubicin for cancer chemotherapy, Drug delivery, 26 (2019) 898-917.

[17] R. Hergt, S. Dutz, R. Müller, M. Zeisberger, Magnetic particle hyperthermia: nanoparticle magnetism and materials development for cancer therapy, Journal of Physics: Condensed Matter, 18 (2006) S2919.

[18] R.E. Rosensweig, Heating magnetic fluid with alternating magnetic field, Journal of magnetism and magnetic materials, 252 (2002) 370-374.

[19] Y. Tang, T. Jin, R.C. Flesch, Numerical temperature analysis of magnetic hyperthermia considering nanoparticle clustering and blood vessels, IEEE Transactions on Magnetics, 53 (2017) 1-6.

[20] L. Wu, J. Cheng, W. Liu, X. Chen, Numerical analysis of electromagnetically induced heating and bioheat transfer for magnetic fluid hyperthermia, IEEE Transactions on Magnetics, 51 (2015) 1-4.

[21] R. Hergt, S. Dutz, Magnetic particle hyperthermia—biophysical limitations of a visionary tumour therapy, Journal of Magnetism and Magnetic Materials, 311 (2007) 187-192.

[22] Q. Wang, Z. Deng, J. Liu, Theoretical evaluations of magnetic nanoparticle-enhanced heating on tumor embedded with large blood vessels during hyperthermia, Journal of Nanoparticle Research, 14 (2012) 974.

[23] M. Bailey, V. Khokhlova, O. Sapozhnikov, S. Kargl, L. Crum, Physical mechanisms of the therapeutic effect of ultrasound (a review), Acoustical Physics, 49 (2003) 369-388.

[24] P. Namakshenas, A. Mojra, Numerical study of non-Fourier thermal ablation of benign thyroid tumor by focused ultrasound (FU), Biocybernetics and Biomedical Engineering, 39 (2019) 571-585.

[25] T. Leslie, R. Ritchie, R. Illing, G. Ter Haar, R. Phillips, M. Middleton, B. Bch, F. Wu, D. Cranston, High-intensity focused ultrasound treatment of liver tumours: post-treatment MRI correlates well with intra-operative estimates of treatment volume, The British journal of radiology, 85 (2012) 1363-1370.

[26] S. Crouzet, J.Y. Chapelon, O. Rouviere, F. Mege-Lechevallier, M. Colombel, H. Tonoli-Catez, X. Martin, A. Gelet, Whole-gland ablation of localized prostate cancer with high-intensity focused ultrasound: oncologic outcomes and morbidity in 1002 patients, European urology, 65 (2014) 907-914.

[27] X.-L. Ren, X.-D. Zhou, J. Zhang, G.-B. He, Z.-H. Han, M.-J. Zheng, L. Li, M. Yu, L. Wang, Extracorporeal ablation of uterine fibroids with high‐intensity focused ultrasound: imaging and histopathologic evaluation, Journal of ultrasound in medicine, 26 (2007) 201-212.

[28] F.P. Curra, P.D. Mourad, V.A. Khokhlova, R.O. Cleveland, L.A. Crum, Numerical simulations of heating patterns and tissue temperature response due to high-intensity focused ultrasound, IEEE transactions on ultrasonics, ferroelectrics, and frequency control, 47 (2000) 1077-1089.

[29] E. Filonenko, V. Khokhlova, Effect of acoustic nonlinearity on heating of biological tissue by high-intensity focused ultrasound, Acoustical Physics, 47 (2001) 468-475.

[30] M.A. Solovchuk, T.W. Sheu, W.-L. Lin, I. Kuo, M. Thiriet, Simulation study on acoustic streaming and convective cooling in blood vessels during a high-intensity focused ultrasound thermal ablation, International Journal of Heat and Mass Transfer, 55 (2012) 1261-1270.

[31] T.W. Sheu, M.A. Solovchuk, A.W. Chen, M. Thiriet, On an acoustics–thermal–fluid coupling model for the prediction of temperature elevation in liver tumor, International Journal of Heat and Mass Transfer, 54 (2011) 4117-4126.

[32] T. Huttunen, M. Malinen, J.P. Kaipio, P.J. White, K. Hynynen, A full-wave Helmholtz model for continuous-wave ultrasound transmission, IEEE transactions on ultrasonics, ferroelectrics, and frequency control, 52 (2005) 397-409.

[33] W.L. Nyborg, Sonically produced heat in a fluid with bulk viscosity and shear viscosity, The Journal of the Acoustical Society of America, 80 (1986) 1133-1139.

[34] F. Henriques Jr, A. Moritz, Studies of thermal injury: I. The conduction of heat to and through skin and the temperatures attained therein. A theoretical and an experimental investigation, The American journal of pathology, 23 (1947) 530.

[35] A. Bhowmik, R. Repaka, S.C. Mishra, K. Mitra, Thermal assessment of ablation limit of subsurface tumor during focused ultrasound and laser heating, Journal of Thermal Science and Engineering Applications, 8 (2016).

[36] P.A. Garcia, R.V. Davalos, D. Miklavcic, A numerical investigation of the electric and thermal cell kill distributions in electroporation-based therapies in tissue, PloS one, 9 (2014) e103083.

[37] D.E. Hilmas, E.L. Gillette, Morphometric analyses of the microvasculature of tumors during growth and after x‐irradiation, Cancer, 33 (1974) 103-110.

[38] J. Pappenheimer, E. Renkin, L. Borrero, Filtration, diffusion and molecular sieving through peripheral capillary membranes: a contribution to the pore theory of capillary permeability, American Journal of Physiology-Legacy Content, 167 (1951) 13-46.

[39] D.J. Schutt, D. Haemmerich, Effects of variation in perfusion rates and of perfusion models in computational models of radio frequency tumor ablation, Medical physics, 35 (2008) 3462-3470.

[40] W. Zhan, W. Gedroyc, X.Y. Xu, Towards a multiphysics modelling framework for thermosensitive liposomal drug delivery to solid tumour combined with focused ultrasound hyperthermia, Biophysics Reports, 5 (2019) 43-59.

[41] T. Balasubramaniam, H. Bowman, Thermal conductivity and thermal diffusivity of biomaterials: A simultaneous measurement technique, (1977).

[42] M. Soltani, M.H. Tehrani, F.M. Kashkooli, M. Rezaeian, Effects of magnetic nanoparticle diffusion on microwave ablation treatment: A numerical approach, Journal of Magnetism and Magnetic Materials, 514 (2020) 167196.

[43] M. Solovchuk, T.W. Sheu, M. Thiriet, Simulation of nonlinear Westervelt equation for the investigation of acoustic streaming and nonlinear propagation effects, The Journal of the Acoustical Society of America, 134 (2013) 3931-3942.

[44] F. Yuan, M. Dellian, D. Fukumura, M. Leunig, D.A. Berk, V.P. Torchilin, R.K. Jain, Vascular permeability in a human tumor xenograft: molecular size dependence and cutoff size, Cancer research, 55 (1995) 3752-3756.

[45] L.E. Gerlowski, R.K. Jain, Microvascular permeability of normal and neoplastic tissues, Microvascular research, 31 (1986) 288-305.

[46] M.H. Gaber, N.Z. Wu, K. Hong, S.K. Huang, M.W. Dewhirst, D. Papahadjopoulos, Thermosensitive liposomes: extravasation and release of contents in tumor microvascular networks, International Journal of Radiation Oncology• Biology• Physics, 36 (1996) 1177-1187.

[47] J.H. Keenan, F.G. Keyes, Thermodynamic properties of steam, (1936).

[48] D.J. Kerr, A.M. Kerr, R.I. Freshney, S.B. Kaye, Comparative intracellular uptake of adriamycin and 4'-deoxydoxorubicin by nonsmall cell lung tumor cells in culture and its relationship to cell survival, Biochemical pharmacology, 35 (1986) 2817-2823.

[49] S. NAGAOKA, S. KAWASAKI, K. SASAKI, T. NAKANISHI, Intracellular uptake, retention and cytotoxic effect of adriamycin combined with hyperthermia in vitro, Japanese Journal of Cancer Research GANN, 77 (1986) 205-211.

[50] L.S. Goodman, Goodman and Gilman's the pharmacological basis of therapeutics, McGraw-Hill New York1996.

[51] E. Saltiel, W. McGuire, Doxorubicin (adriamycin) cardiomyopathy—a critical review, Western Journal of Medicine, 139 (1983) 332.

[52] Y.-M.F. Goh, H.L. Kong, C.-H. Wang, Simulation of the delivery of doxorubicin to hepatoma, Pharmaceutical Research, 18 (2001) 761-770.

[53] A. Gabizon, R. Catane, B. Uziely, B. Kaufman, T. Safra, R. Cohen, F. Martin, A. Huang, Y. Barenholz, Prolonged circulation time and enhanced accumulation in malignant exudates of doxorubicin encapsulated in polyethylene-glycol coated liposomes, Cancer research, 54 (1994) 987-992.

[54] T. Tagami, M.J. Ernsting, S.-D. Li, Optimization of a novel and improved thermosensitive liposome formulated with DPPC and a Brij surfactant using a robust in vitro system, Journal of controlled release, 154 (2011) 290-297.

[55] M.N. Centelles, M. Wright, P.-W. So, M. Amrahli, X.Y. Xu, J. Stebbing, A.D. Miller, W. Gedroyc, M. Thanou, Image-guided thermosensitive liposomes for focused ultrasound drug delivery: using NIRF-labelled lipids and topotecan to visualise the effects of hyperthermia in tumours, Journal of Controlled Release, 280 (2018) 87-98.

[56] A. Gasselhuber, M.R. Dreher, F. Rattay, B.J. Wood, D. Haemmerich, Comparison of conventional chemotherapy, stealth liposomes and temperature-sensitive liposomes in a mathematical model, PloS one, 7 (2012) e47453.

[57] D. Needham, M.W. Dewhirst, The development and testing of a new temperature-sensitive drug delivery system for the treatment of solid tumors, Advanced drug delivery reviews, 53 (2001) 285-305.

[58] F.M. Kashkooli, M. Soltani, M. Rezaeian, C. Meaney, M.-H. Hamedi, M. Kohandel, Effect of vascular normalization on drug delivery to different stages of tumor progression: In-silico analysis, Journal of Drug Delivery Science and Technology, 60 (2020) 101989.

[59] P. Vaupel, F. Kallinowski, P. Okunieff, Blood flow, oxygen and nutrient supply, and metabolic microenvironment of human tumors: a review, Cancer research, 49 (1989) 6449-6465.

[60] A. Gasselhuber, M.R. Dreher, A. Partanen, P.S. Yarmolenko, D. Woods, B.J. Wood, D. Haemmerich, Targeted drug delivery by high intensity focused ultrasound mediated hyperthermia combined with temperature-sensitive liposomes: computational modelling and preliminary in vivo validation, International Journal of Hyperthermia, 28 (2012) 337-348.

[61] P.S. Tofts, G. Brix, D.L. Buckley, J.L. Evelhoch, E. Henderson, M.V. Knopp, H.B. Larsson, T.Y. Lee, N.A. Mayr, G.J. Parker, Estimating kinetic parameters from dynamic contrast‐enhanced T1‐weighted MRI of a diffusable tracer: standardized quantities and symbols, Journal of Magnetic Resonance Imaging: An Official Journal of the International Society for Magnetic Resonance in Medicine, 10 (1999) 223-232.

[62] D.M. Brizel, B. Klitzman, J.M. Cook, J. Edwards, G. Rosner, M.W. Dewhirst, A comparison of tumor and normal tissue microvascular hematocrits and red cell fluxes in a rat window chamber model, International Journal of Radiation Oncology* Biology* Physics, 25 (1993) 269-276.

[63] F. Yuan, M. Leunig, D.A. Berk, R.K. Jain, Microvascular permeability of albumin, vascular surface area, and vascular volume measured in human adenocarcinoma LS174T using dorsal chamber in SCID mice, Microvascular research, 45 (1993) 269-289.

[64] N.Z. Wu, B. Klitzman, G. Rosner, D. Needham, M.W. Dewhirst, Measurement of material extravasation in microvascular networks using fluorescence video-microscopy, Microvascular research, 46 (1993) 231-253.

[65] R.K. Jain, Transport of molecules in the tumor interstitium: a review, Cancer research, 47 (1987) 3039-3051.

[66] E.A. Swabb, J. Wei, P.M. Gullino, Diffusion and convection in normal and neoplastic tissues, Cancer research, 34 (1974) 2814-2822.

[67] L.J. Nugent, R.K. Jain, Extravascular diffusion in normal and neoplastic tissues, Cancer research, 44 (1984) 238-244.

[68] M.B. Wolf, P.D. Watson, D. Scott 2nd, Integral-mass balance method for determination of solvent drag reflection coefficient, American Journal of Physiology-Heart and Circulatory Physiology, 253 (1987) H194-H204.

[69] C. Liu, J. Krishnan, X.Y. Xu, Investigating the effects of ABC transporter-based acquired drug resistance mechanisms at the cellular and tissue scale, Integrative Biology, 5 (2013) 555-568.

[70] L.Z. Benet, P. Zia-Amirhosseini, Basic principles of pharmacokinetics, Toxicologic pathology, 23 (1995) 115-123.

[71] K.A. Rodvold, D.A. Rushing, D.A. Tewksbury, Doxorubicin clearance in the obese, Journal of Clinical Oncology, 6 (1988) 1321-1327.

[72] A. Gasselhuber, M.R. Dreher, A. Negussie, B.J. Wood, F. Rattay, D. Haemmerich, Mathematical spatio-temporal model of drug delivery from low temperature sensitive liposomes during radiofrequency tumour ablation, International Journal of Hyperthermia, 26 (2010) 499-513.

[73] T. Stylianopoulos, E.-A. Economides, J.W. Baish, D. Fukumura, R.K. Jain, Towards optimal design of cancer nanomedicines: Multi-stage nanoparticles for the treatment of solid tumors, Annals of biomedical engineering, 43 (2015) 2291-2300.

[74] K. Murase, J. Oonoki, H. Takata, R. Song, A. Angraini, P. Ausanai, T. Matsushita, Simulation and experimental studies on magnetic hyperthermia with use of superparamagnetic iron oxide nanoparticles, Radiological physics and technology, 4 (2011) 194-202.

[75] S. Maenosono, S. Saita, Theoretical assessment of FePt nanoparticles as heating elements for magnetic hyperthermia, IEEE transactions on magnetics, 42 (2006) 1638-1642.

[76] M. Minbashi, A.A. Kordbacheh, A. Ghobadi, V.V. Tuchin, Optimization of power used in liver cancer microwave therapy by injection of Magnetic Nanoparticles (MNPs), Computers in Biology and Medicine, (2020) 103741.
